# Butyrophilin-like 2 regulates site-specific adaptations of intestinal γδ intraepithelial lymphocytes

**DOI:** 10.1038/s42003-021-02438-x

**Published:** 2021-07-26

**Authors:** Casandra Panea, Ruoyu Zhang, Jeffrey VanValkenburgh, Min Ni, Christina Adler, Yi Wei, Francisca Ochoa, Jennifer Schmahl, Yajun Tang, Chia-Jen Siao, William Poueymirou, Jennifer Espert, Wei Keat Lim, Gurinder S. Atwal, Andrew J. Murphy, Matthew A. Sleeman, Zaruhi Hovhannisyan, Sokol Haxhinasto

**Affiliations:** Regeneron Pharmaceuticals Inc., Tarrytown, NY USA

**Keywords:** Mucosal immunology, Innate immunity

## Abstract

Tissue-resident γδ intraepithelial lymphocytes (IELs) orchestrate innate and adaptive immune responses to maintain intestinal epithelial barrier integrity. Epithelia-specific butyrophilin-like (Btnl) molecules induce perinatal development of distinct Vγ TCR^+^ IELs, however, the mechanisms that control γδ IEL maintenance within discrete intestinal segments are unclear. Here, we show that Btnl2 suppressed homeostatic proliferation of γδ IELs preferentially in the ileum. High throughput transcriptomic characterization of site-specific *Btnl2*-KO γδ IELs reveals that Btnl2 regulated the antimicrobial response module of ileal γδ IELs. Btnl2 deficiency shapes the TCR specificities and TCRγ/δ repertoire diversity of ileal γδ IELs. During DSS-induced colitis, *Btnl2*-KO mice exhibit increased inflammation and delayed mucosal repair in the colon. Collectively, these data suggest that Btnl2 fine-tunes γδ IEL frequencies and TCR specificities in response to site-specific homeostatic and inflammatory cues. Hence, Btnl-mediated targeting of γδ IEL development and maintenance may help dissect their immunological functions in intestinal diseases with segment-specific manifestations.

## Abstract (web summary)

Panea et al showed that epithelia-specific butyrophilinlike 2 (Btnl2) suppressed homeostatic proliferation of γδ intraepithelial lymphocytes (IELs) preferentially in the ileum and used high throughput transcriptomic characterization of Btnl2-deficient γδ IELs to demonstrate that Btnl2 impacts γδ TCR specificities and repertoire diversity of ileal γδ IELs. In addition, they showed that Btnl2-deficient mice exhibited increased inflammation and delayed mucosal repair in the colon, suggesting that it plays a key immunological function in intestinal diseases.

## Introduction

Tissue-resident intraepithelial lymphocytes (IELs) represent a heterogenous population of antigen-experienced immune cells in the intestinal epithelium that are involved in the maintenance of gut homeostasis^1,2^. In particular, IELs expressing αβ T cell receptors (TCRs) are poised for mounting pathogen-specific memory responses, while those possessing γδ TCRs strengthen tight junctions and orchestrate innate and adaptive immunity during homeostasis, inflammation, and infection^1–6^. Interactions between intestinal epithelial cells (IECs) and γδ IELs influence IEL development and function^7,8^. Notably, recent studies emphasized that anatomical segregation could drive gut segment-specific immunity^9–11^, including functionally distinct γδ IEL immune responses to chemically-induced and pathogen-induced epithelial injury^5,12–14^. However, the mechanisms that regulate γδ IEL development and maintenance in response to the local antigenic environment remain poorly understood.

Recent studies provided some evidence that IEC-specific butyrophilin-like (Btnl) molecules induce perinatal expansion and maturation of distinct Vγ TCR^+^ IELs^5,7,15–17^. Indeed, intestinal γδ IELs predominantly express Vγ7 in mice and Vγ4 in humans that persist throughout the life of the host^5,7^. γδ IELs continuously sample both self and bacterial antigens from the local environment to customize their TCR specificities^5,16^. Moreover, the contributions of Btnl molecules to shaping γδ TCR repertoire diversity and regulating the distribution and function of γδ IEL subsets across intestinal compartments^18,19^ remain to be elucidated and may inform our understanding of the compartmentalized immune responses observed in the intestine^9,11,14^.

Btn/Btnl proteins are members of B7 immunoglobulin-superfamily and analogous to other costimulatory and coinhibitory molecules (e.g., CD80, CD86, PDL1, and PDL2) have been shown to modulate αβ T cell immune functions, including inhibition of CD4^+^ T and CD8^+^ T cell activation, proliferation and cytokine production, induction of regulatory T (Treg) cells and blockade of antigen-specific proinflammatory responses^20–27^. Btnl2, a member of the Btnl family, has been shown to induce Treg differentiation and suppress T cell activation and proliferation in vitro^25^. Accordingly, *Btnl2*-KO chimera mice displayed increased susceptibility in a mouse model of experimental cerebral malaria and higher frequencies of peripheral CD4^+^ T and CD8^+^ T cells indicating a potential role for Btnl2 in dampening infection-elicited T cell immune responses in vivo^28^. *Btnl2* is highly enriched in villous IECs across different intestinal compartments and its expression is reported to be altered by inflammatory cues such as epithelial injury and tumor burden^21,29–32^. In particular, *Btnl2* mRNA levels were increased in the colon of *Mdr1a*-KO colitic mice and decreased in human colon tumors^29,32^. Furthermore, truncating single nucleotide polymorphisms (SNPs) of *BTNL2* were associated with ulcerative colitis (UC) and chronic sarcoidosis, independent of linkage disequilibrium (LD) with HLA^21,29–31^. The role of *Btnl2* in regulating intestinal immune responses during homeostasis and inflammation and, particularly, in the induction and maintenance of intestinal γδ IELs has not yet been addressed.

Here, we report the generation and characterization of *Btnl2* knockout mice and identify a role for Btnl2 in regulating the frequencies and phenotype of γδ IELs preferentially in the ileum at a steady state. We found that γδ IELs derived from the ileum, but not duodenum, of *Btnl2-*KO mice possess a dysregulated antibacterial response module. By integrating RNA and single-cell TCR expression data we identified distinct transcriptional signatures and greater TCR repertoire diversity in ileal *Btnl2-*KO γδ IELs. Upon DSS challenge, *Btnl2-*KO mice displayed enhanced colonic, but not ileal, intestinal inflammation, and delayed mucosal repair. Collectively, our findings suggest that context-dependent Btnl2 expression fine-tunes intestinal immune responses to protect against epithelial injury.

## Results

### *Btnl2* is preferentially expressed in small intestinal epithelial cells

**Fig. 1:**
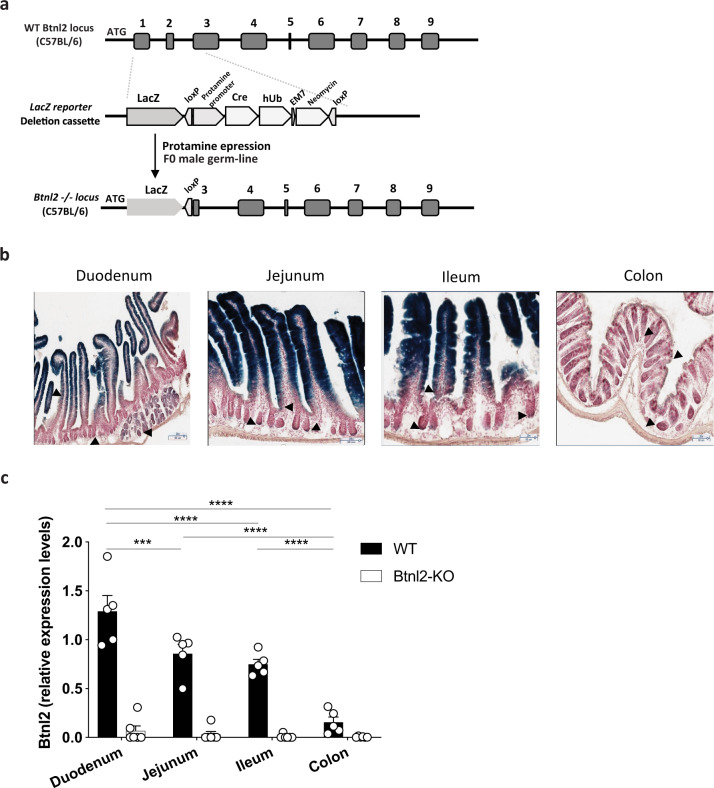
Btnl2 is preferentially expressed in small intestinal epithelial cells. **a** Schematic representation of the WT and targeted locus of Btnl2−/− mice. hUb, human Ubiquitin promoter. **b** Beta-galactosidase and Neutral Red counterstaining in cryosections of segments of the small intestine (duodenum, jejunum, and ileum) and colon of 15-week-old Btnl2-KO mice. Arrowheads indicate regions of duodenal glands and duodenal, jejunal, ileal, and colonic crypts and villi with weak Btnl2-LacZ expression. Magnification is 20×; scale bar is 50 μm. **c** mRNA expression of Btnl2 in intestinal epithelial cells from different segments of small intestine and colon of cohoused 7-week-old Btnl2-KO and WT mice (*n* = 5, each), normalized to β2m. Error bars represent mean ± SEM. Significance is measured using multiple unpaired *t*-tests assuming similar SD, *p < 0.05, **p < 0.005, ***p < 0.0005, ****p < 0.0001, significantly different from WT mice.

To determine the expression pattern of *Btnl2* in the intestine during homeostasis, we generated *Btnl2*-LacZ knock-in mice henceforth referred to as *Btnl2-*KO mice (Fig. 1a). Consistent with previous observations^21,29^, *Btnl2* was predominantly expressed in terminally differentiated IECs of the small intestine (Fig. 1b). Importantly, unlike *Btnl1*, *Btnl4,* and *Btnl6*^7^, *Btnl2* expression was detected in duodenal Brunner’s glands and duodenal, jejunal, ileal, and colonic crypts, suggesting potential divergent roles of different Btnls dictated by their region-specific expression patterns (Fig. 1b). To further validate our observations, we measured *Btnl2* mRNA levels in IECs derived from terminally differentiated enterocytes isolated from the duodenum, jejunum, ileum, and distal colon. *Btnl2* was highly enriched in duodenal IECs with a descending proximal-to-distal gradient, such that *Btnl2* expression in IECs isolated from the colon was ~5-fold lower than in the ileum of WT mice (Fig. 1c). Similarly, *BTNL2* transcripts were detected in the small intestine, but not colon samples pooled from healthy human tissues^30^.

Given the close proximity of the *Btnl2* gene to the H2 locus and other *Btnls*^33^, we investigated the expression levels of several adjacent genes in the IEC fraction isolated from duodenum, jejunum, and ileum of *Btnl2-*KO and WT mice by bulk RNA-sequencing. We did not observe any significant changes in *H2-Aa, H2-Ab1, H2-Eb1, Tap1/2, BC051142, Btnl4, Btnl5, Btnl6, Notch4,* and *Ppt2* gene expression levels across different segments of the small intestine, albeit *Btnl2-*KO mice displayed a trend towards decreased levels of *Btnl1* (Supplementary Figure 1), indicating no significant coregulation of *Btnl2* with adjacent genes near the *H2* locus. Altogether, our data confirm preferential expression of *Btnl2* in terminally differentiated enterocytes across different segments of the small intestine suggesting a compartment-specific function of Btnl2.

### *Btnl2-*KO mice display increased frequencies of γδ IELs preferentially in the ileum

Under homeostatic conditions, *Btnl2-*KO mice did not exhibit any adverse intestinal pathology, as determined by body weight loss, increased epithelial sloughing, and pro-inflammatory cytokines (Supplementary Figure 2a, b). In addition, we did not observe any significant changes in genes associated with differentiation and maturation of IECs^34–41^, suggesting that IEC development and maintenance are not altered in unchallenged *Btnl2-*KO mice (Supplementary Figure 2c).

**Fig. 2:**
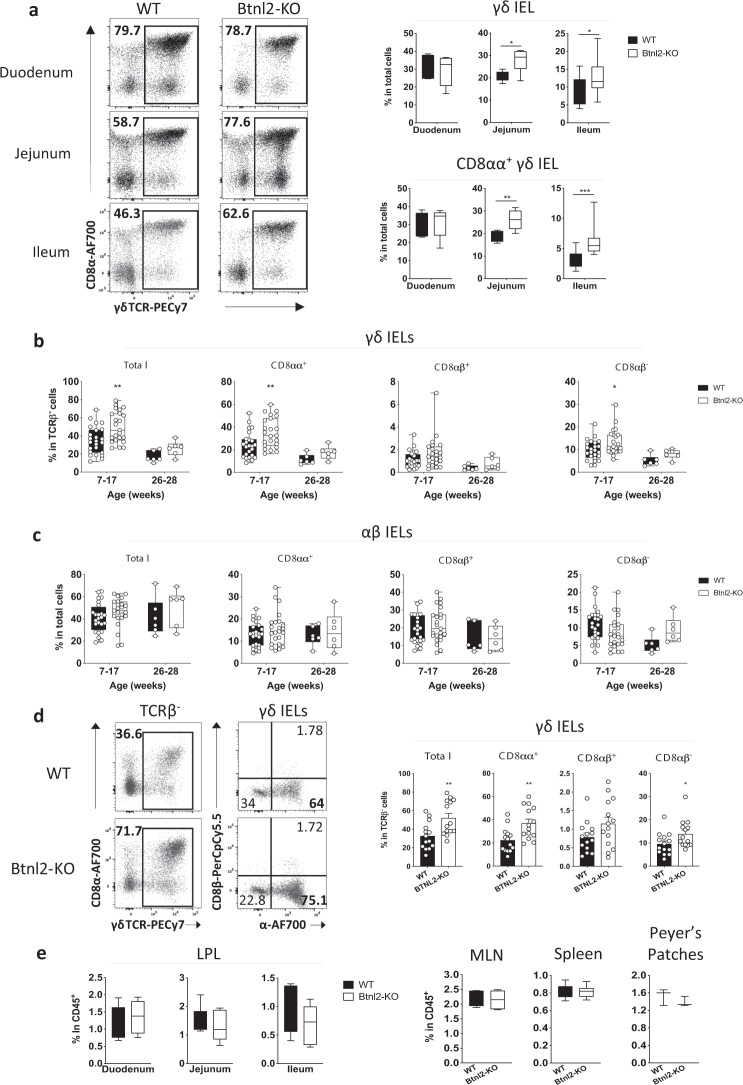
Btnl2-KO mice display increased frequencies of γδ IELs in the ileum. **a** Different segments of the small intestine were collected from cohoused 7–17-week-old Btnl2-KO and WT mice (*n* = 3–8, each) and processed for flow cytometry. Left panel-representative flow cytometry plots of γδ IELs in the duodenum, jejunum, and ileum of cohoused 7-week-old Btnl2-KO and WT littermates. Displayed plots are gated on live TCRαβ- cells. Right panel-frequencies of γδ IELs and CD8αα + γδ IELs from 7–17-week-old Btnl2-KO and WT littermates. **b** Frequencies of ileal γδ IELs at different ages (*n* = 6–23 mice/group). Error bars represent mean ± SEM. Significance is measured by 2-way ANOVA with Sidak’s multiple comparison test, *p < 0.05, **p < 0.005. **c** Frequencies of ileal αβ IELs at different ages (*n* = 6–23 mice/group). Error bars represent mean ± SEM. Significance is measured by 2-way ANOVA. **d** Ileum was collected from cohoused Btnl2-KO and WT littermates of different ages and intestinal intraepithelial lymphocytes (IELs) were isolated and processed for flow cytometry. Left panel-representative flow cytometry plots of γδ IELs in the ileum of 12–17-week-old Btnl2-KO and WT littermates. Right panel-frequencies of ileal γδ IELs from 12–17-week-old Btnl2-KO and WT littermates. Data are pooled from 3 independent experiments with 3–6 mice/group. Error bars represent mean ± SEM. Significance is measured using unpaired *t*-tests assuming similar SD, *p < 0.05, **p < 0.005, significantly different from WT mice. **e** Frequencies of γδ T cells in lamina propria (LP), mesenteric lymph nodes (MLN), spleen, and Peyer’s Patches of 7–17-week-old Btnl2-KO and WT littermates (*n* = 3–6 mice/group).

In light of the developing paradigm implicating members of Btnl/BTN/BTNL family in shaping the γδ T cell compartment^5,7,16,17,42,43^ and intrigued by the selective expression pattern of Btnl2 in different segments of the small intestine, we postulated *Btnl2* deficiency might impact the maintenance of γδ IEL subsets in different segments of the small intestine. To this end, we isolated IELs from the duodenum, jejunum, and ileum of adult *Btnl2-*KO and WT mice. Consistent with the previous observations^14,18,19^, γδ IELs were found at ~3-fold higher frequency in the duodenum compared to the ileum of WT mice (30.7% vs. 8.62% in total cells, Fig. 2a-right panel), however we observed that compared to WT littermates, *Btnl2-*KO mice displayed a 30–40% increase in the frequency of γδ IELs in the jejunum (27.1% vs. 20.1%) and ileum (12.8% vs. 8.6%), but not duodenum (29.4% vs. 30.7%) (Fig. 2a). Notably, this increase was observed predominantly in ileal CD8αα^+^ γδ IELs suggesting that Btnl2 may suppress their proliferative capacity in situ (Fig. 2a). As the number of Vγ7^+^ IELs have been reported to plateau in 11–16-week-old young adults^7^, we investigated whether the observed effect of Btnl2 on the percentage of ileal γδ IELs changed as mice approached middle adulthood. Previous work suggested that ileal γδ IEL frequencies, including CD8αα^+^ γδ IELs, remain steady past 6 months of age in WT mice^44^. In contrast, we observed that γδ IEL frequency was reduced by 50% at 6 months of age in WT mice in our facility (39.5% vs. 17.1% in total γδ IELs) (Fig. 2b, d**)**. Conversely, αβ IEL frequency remained relatively unchanged at 6 months of age suggesting that αβ IELs actively maintain their levels, possibly through in situ expansion (Fig. 2c). We found that γδ IELs were significantly increased in the ileum of young adult (up to 4 months old) *Btnl2-*KO compared to WT mice (36.9% vs. 22.3% in CD8αα^+^ γδ IELs), while mature adult mice displayed similar levels of γδ IELs (Fig. 2b, d). αβ IELs were not significantly altered in *Btnl2*-KO mice during this time frame (Fig. 2c). As such, the Btnl2 effect in young adult mice suggests it plays a role in γδ IEL maintenance under homeostatic conditions. Notably, γδ T cells were observed at comparable frequencies in the lamina propria (LP) of the duodenum, jejunum, and ileum, mesenteric lymph nodes (mLN), and Peyer’s Patches (PP) of *Btnl2-*KO and WT mice indicating that Btnl2 exerts its function specifically on γδ CD8αα^+^ IELs (Fig. 2e).

Prior studies had shown that recombinant Btnl2 can inhibit mLN CD4^+^ T cell proliferation and promote Treg cell differentiation under certain activation conditions in vitro^21,22,25,29^. Nevertheless, we observed similar frequencies of CD4^+^ T cells and FoxP3^+^ Tregs in the ileal LP, mLNs, and PPs of *Btnl2-*KO mice. In addition, *Btnl2-*KO mice exhibited comparable immune cell profiles across different tissues emphasizing the specificity and localized effect of Btnl2 effects in the intestine on jejunal and ileal γδ IELs (Supplementary Figure 3a–c).

### *Btnl2* suppresses proliferation of jejunal/ileal γδ IELs

To investigate the effects that *Btnl2* exerted on jejunal and ileal γδ IELs, we revisited its ability to suppress T cell proliferation. As Btnl2 inhibitory function is dependent on concurrent TCR stimulation and ligation with the putative Btnl2 receptor on CD4^+^ T cells in vitro^21,22,25,29^, we activated CFSE-labeled CD4^+^ T cells in the presence of equimolar concentrations of plate-bound Btnl2*-*mFc, Pdl1*-*mFc, Pdl2*-*mFc, or mFc (Supplementary Figure 4a). After 72 h of culture, we found that recombinant Btnl2 potently suppressed proliferation and activation of CD4^+^ T cells similarly to *Pdl1* and *Pdl2*, as evidenced by CFSE dilution and greater than 40% decrease in cytokine production (Supplementary Figure 4b, c). In line with previous reports^25^, CD28 co-stimulation rescued production of TNFα and IFNγ, but not IL-2 (Supplementary Figure 4c), which also coincided with ~50% decrease in Btnl2 binding to its putative receptor on activated CD4^+^ T cells (Supplementary Figure 4d).

**Fig. 3:**
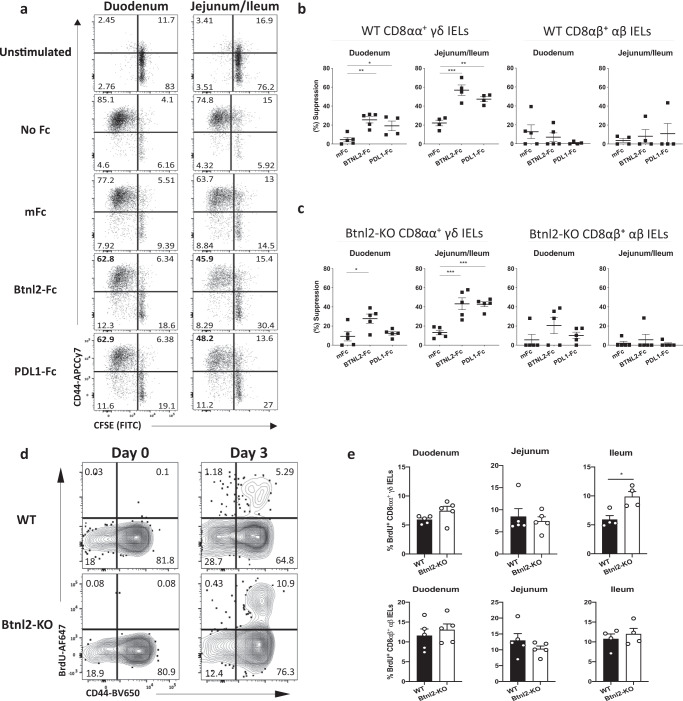
Btnl2 suppresses proliferation of jejunal/ileal γδ IELs. IELs were isolated from duodenum and jejunum/ileum of cohoused 12-week-old Btnl2-KO and WT mice (*n* = 4–5, each), labeled with CFSE and stimulated with α-CD3 and different Fc fusions in the presence of rh IL-2, rmIL7, rm IL15 for 84 h. Supernatants from the cell cultures were collected and cells were processed for flow cytometry. **a** Representative flow cytometry plots of duodenal and jejunal/ileal WT CD8αα + γδ IELs following 84 h of culture in the presence of equimolar concentrations of Btnl2-Fc and control mFc fusion proteins. **b**, **c** Suppression of proliferation calculated as the percent difference between the proliferation in the presence of a specific Fc fusion and no Fc fusion, relative to the proliferation in the absence of Fc fusion. **b** Suppression of proliferation of duodenal and jejunal/ileal WT CD8αα + γδ IELs and CD8αβ + αβ IELs. **c** Suppression of proliferation of duodenal and jejunal/ileal Btnl2-KO CD8αα + γδ IELs and CD8αβ + αβ IELs. Error bars represent mean ± SEM. Significance is measured using one-way ANOVA, *p < 0.05, **p < 0.005, ***p < 0.001, ****p < 0.0001, significantly different from mFc fusion protein control. **d**, **e** Cohoused 11-week-old Btnl2-KO and WT mice (*n* = 4–5, each) were given BrdU at 0.8 mg/mL ad libitum in drinking water for 3 days. BrdU incorporation was measured by intranuclear staining of γδ IELs from different segments of the small intestine over time. **d** Representative flow cytometry plots of BrdU incorporation in ileal CD8αα + γδ IELs. **e** BrdU incorporation in CD8αα + γδ IELs and CD8αβ + αβ IELs across different segments. Error bars represent mean ± SEM. Significance is measured using unpaired *t*-tests assuming similar SD, *p < 0.05, **p < 0.005, ***p < 0.0005, ****p < 0.0001, significantly different from WT.

We next sought to determine whether Btnl2 suppresses the proliferation of γδ IELs in vitro (Supplementary Figure 5a). To obtain comparable numbers to those from the duodenum, we pooled IELs from the jejunum and ileum. Interestingly, duodenal CD8αα^+^ γδ IELs showed greater proliferative capacity compared to their jejunal/ileal counterparts indicating that duodenum and jejunal/ileal γδ IELs may require different TCR and/or cytokine stimulation (Fig. 3a). Recombinant Btnl2 and Pdl1 potently inhibited the proliferation of both duodenal and jejunal/ileal CD8αα^+^ γδ IELs (Fig. 3a, b). However, Btnl2 suppressive effect was 2-fold higher on jejunal/ileal CD8αα^+^ γδ IEL proliferation compared to one observed for duodenal CD8αα^+^ γδ IELs (Fig. 3b). Importantly, recombinant Btnl2 failed to inhibit the proliferation of duodenal or jejunal/ileal CD8αβ^+^ αβ IELs, suggesting that the Btnl2 putative receptor may not be present on these cells (Fig. 3b). Contrary to previous reports suggesting stronger responsiveness of *Btnl1-*KO γδ IELs to α-CD3 stimulation^7^, we found that duodenal and jejunal/ileal *Btnl2-*KO γδ and αβ IELs exhibited equal proliferative capacity compared to their WT counterparts, indicating that *Btnl2* deficiency did not impair the ability of IELs to respond to TCR and cytokine stimulation (Supplementary Figure 5b). Moreover, recombinant Btnl2 similarly inhibited the proliferation of *Btnl2-*KO and WT jejunal/ileal γδ IELs in vitro (Fig. 3c). *Btnl2-*KO and WT jejunal/ileal γδ IELs also showed comparable expression profiles of coinhibitory receptors (e.g. PD1) and markers associated with tissue residence, maturation, and activation (e.g. CD69, CD44, CD27, and CD122)^7,45^ (Supplementary Figure 5c). Altogether, these data indicate that Btnl2 preferentially suppresses jejunal/ileal CD8αα^+^ γδ IEL proliferation.

To determine the proliferative capacity of *Btnl2-*KO γδ IELs in vivo, we measured BrdU incorporation in γδ IELs. As previously reported^46^, high BrdU incorporation was observed in WT CD8αα^+^ γδ IELs by day 3 (Fig. 3d, e). Notably, ileal *Btnl2-*KO γδ IELs incorporated BrdU ~2-fold more than their WT counterparts (9.86 $\pm$ 1.52 vs. 5.87 $\pm$ 1.18), emphasizing the enhanced proliferative capacity of γδ IELs in the absence of *Btnl2* (Fig. 3d, e). Overall, these observations indicate that Btnl2 may serve as a γδ immune checkpoint molecule by limiting the expansion of mature γδ IELs in the ileum and, potentially, regulate their effector responses during the normal epithelial lifespan.

### Ileal *Btnl2-*KO γδ IELs display a subdued antibacterial response module

**Fig. 4:**
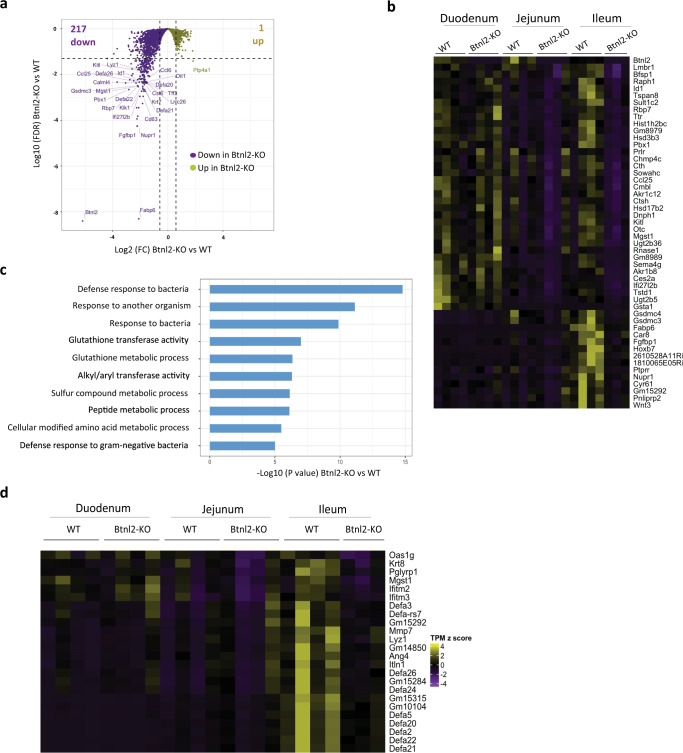
Ileal Btnl2-KO γδ IELs display an altered antibacterial response module compared to ileal WT γδ IELs. γδ IELs from duodenum, jejunum, and ileum of cohoused 11-week-old Btnl2-KO and WT littermates (*n* = 3–4/genotype, each a pool of 2 mice) were sort-purified as CD45 + TCRβ-TCRγδ + cells. RNA sequencing was performed, and gene set enrichment analysis using NextBio. Gene ontology (GO) was employed to identify GO biological processes differentially enriched in Btnl2-KO and WT γδ IELs. **a** Volcano plot displaying genes differentially regulated between ileal Btnl2-KO and WT γδ IELs. The Horizontal dashed line indicates FDR = 0.05 and a vertical dashed line indicates |Fold Change | = 1.5. **b** Hierarchical clustering of top 50 differentially expressed genes between ileal Btnl2-KO and WT γδ IELs; duodenal and jejunal Btnl2-KO and WT γδ IELs were also included as a comparison. **c** Gene ontology enrichment analysis of most significantly impaired biological processes in ileal Btnl2-KO γδ IELs compared to ileal WT γδ IELs. **d** Hierarchical clustering of top dysregulated antibacterial response genes in ileal Btnl2-KO and WT γδ IELs within the combined top 3 GO processes; duodenal and jejunal Btnl2-KO and WT γδ IELs were also included as a comparison.

To gain insights into the impact of *Btnl2* deficiency on γδ IEL cytolytic potential, we performed transcriptomic analysis of γδ IELs enriched from duodenum, jejunum, and ileum of 11-week-old cohoused *Btnl2-*KO and WT mice at steady-state. Over 200 genes were significantly downregulated (FDR < 0.05 and fold change > 1.5) in ileal *Btnl2-*KO γδ IELs compared to their WT counterparts (Fig. 4a), whereas only *Btnl2* was significantly decreased in duodenal and jejunal *Btnl2-*KO γδ IELs (Fig. 4b). The top 50 downregulated genes included signaling molecules (e.g. *Raph1, Cyr61*, *Tspan8*), transcriptional regulators of cell proliferation and apoptosis (e.g. *Id1*, *Pbx1*, *Nupr1*, *Hoxb7*), growth factors (e.g. *Kitl*, *Wnt3*, *Fgfbp1*), antimicrobial molecules (e.g. *Ifi27l2b, Ccl25*, *Gsdmc3/4*) and different classes of metabolic molecules (e.g. aminoacid-*Mgst1*, lipid-*Fabp6*, *Pnliprp2*, sulfur-*Sult1c2*, *Cth*, carbonic anhydrases-*Car8*) (Fig. 4a, b). Collectively, these observations hint at some decrease in the metabolic function of ileal *Btnl2*-KO γδ IELs compared to ileal WT γδ IELs.

In line with these findings, gene ontology enrichment analysis revealed that the most significantly dysregulated biological processes centered around bacterial tolerance and clearance, emphasizing that ileal *Btnl2-*KO γδ IELs display an impaired ability to secrete antimicrobial molecules at a steady-state (Fig. 4c). Interferon-induced molecules and several members of the α-defensin antimicrobial peptide family were found among the genes significantly downregulated in ileal *Btnl2-*KO γδ IELs compared to their WT counterparts (Fig. 4d). Hence, our findings indicate that γδ IELs in the ileum, but not duodenum or jejunum, may be specialized in secreting antibacterial molecules in response to local microbial antigens.

### Single-cell TCR sequencing highlights greater repertoire diversity in *Btnl2-*KO γδ IELs

**Fig. 5:**
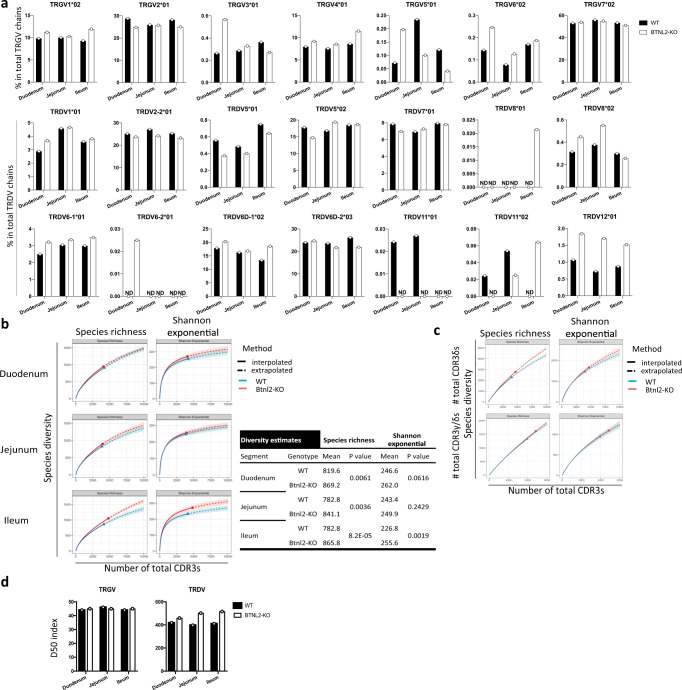
Ileal Btnl2-KO γδ IELs exhibit more diverse TRGV repertoire compared to ileal WT γδ IELs. γδ IELs from the duodenum, jejunum, and the ileum of cohoused 11-week-old Btnl2-KO and WT littermates (*n* = 3–4/genotype, a pool of 2 mice, each) were sort-purified as CD45 + TCRβ-TCRγδ + cells. Two-thirds of each sample were processed for deep bulk RNA sequencing and one-third of each sample was pooled per genotype per segment and used for single-cell sorting and single-cell TCR sequencing. γδ IELs from duodenum, jejunum, and ileum of cohoused 11-week-old Btnl2-KO and WT littermates (*n* = 8 mice, each) were single-cell sorted and single-cell TCR sequencing analysis of TCR Vγ and TCR Vδ chain usage and CDR3 aminoacid sequences was performed. **a** TRGV*J and TRDV*J gene usage in Btnl2-KO and WT γδ IELs. ND not detected. **b** Top-Diversity estimates for TRG only CDR3 aminoacid sequences in Btnl2-KO and WT γδ IELs. Shaded areas indicate the 95% confidence interval by 50 bootstrap replicates. Bottom-TRG diversity estimates at interpolation point 3500, where *p* value is derived from t-test based on 50 bootstrap replicates. **c** Diversity estimates for TRD only and paired TRG and TRD CDR3 aminoacid sequences, respectively, in ileal Btnl2-KO and WT γδ IELs. Shaded areas indicate the 95% confidence interval by 50 bootstrap replicates. **d** Diversity 50 (D50) index represented as the number of top unique clones that comprise 50% of the TRGV and TRDV repertoires, respectively, normalized to the total number of unique clones of duodenal, jejunal and ileal Btnl2-KO and WT γδ IELs.

To determine whether the downregulated antibacterial response module observed in ileal *Btnl2-*KO γδ IELs related to an altered γ/δ TCR repertoire, we performed unbiased single-cell TCR sequencing on duodenal, jejunal and ileal γδ IELs from Btnl2-KO and WT mice. In total, we sequenced 28,679 cells and reassembled 24,961 productive γ chains and 24,515 productive δ chains. Among all sequenced cells, 17,260 cells (60.2%) had paired γ and δ chains. We found that *TRGV* and *TRDV* gene usage was comparable between *Btnl2-*KO and WT γδ IELs in the duodenum, jejunum, and ileum (Fig. 5a). *TRGV7* gene usage averaged 50% of TCR γ chains, consistent with the previous reports^7^, whereas *TRDV2-2, TRDV5, TRDV6D-1*, and *TRDV6D-2* genes were equally represented and their combined gene usage surpassed 80% of TCR δ chains. Ileal *Btnl2*-KO γδ IELs used the *TRGV7* gene less frequently than their WT counterparts (51.0% vs. 53.2%), however, they employed the less common *TRGV4* gene more frequently (11.5% vs. 8.6%; Pearson’s chi-squared test, *p* = 1.78 × 10^−7^). Similarly, ileal *Btnl2*-KO γδ IELs showed reduced usage of *TRDV2-2* and *TRDV6D-2* genes compared to their WT counterparts (23.2% vs. 25.3%; 21.9% vs. 26.2%, respectively), whereas usage of *TRDV6D-1* gene (18.6% vs. 13.4%) and of the less employed *TRDV12* gene (1.52% vs. 0.87%) increased.

To establish TCR γ chain and δ chain clonotypes, we identified TCR γ and δ sequences encoded by the same V gene and J gene segments with identical aminoacid sequences in the third complementarity determining regions (CDR3). Using the R iNext package^47^, we computed two metrics (Species richness and Shannon diversity) to estimate TCR diversity for each sample. Overall, ileal *Btnl2*-KO γδ IELs had consistently higher TCR γ chain diversity than WT γδ IELs by both measurements in both interpolated and extrapolated data. For example, we randomly sampled 3500 γ chains from each sample by 50 bootstrap replications and observed that the mean Shannon diversity of ileal *Btnl2*-KO γ chains was 255.6, significantly higher than 226.8 in WT (*p* = 0.0019, *T*-test) (Fig. 5b). Duodenal and jejunal *Btnl2*-KO γδ IELs also showed a trend towards higher diversity in TCR γ chain compared to WT γδ IELs but the difference was marginal when contrasted to their ileal counterparts (*p* = 0.0616 and 0.2429, respectively for Shannon diversity when sampling 3500 γ chains, Fig. 5b). Although ileal *Btnl2*-KO γδ IELs had increased diversity in TCR δ chains (Fig. 5c), *Btnl2*-KO γδ IELs isolated from all three segments displayed an increased frequency of unique clonotypes that comprised 50% of the TRDV repertoire (duodenum: 19.0% vs. 17.6 %; jejunum: 20.2% vs. 18.6%; and ileum: 18.5% vs. 18%) (Fig. 5d). In contrast, the overall repertoire diversity of paired γ/δ chains was marginally altered in ileal *Btnl2*-KO γδ IELs (Fig. 5c). Collectively, these results suggested that the ileal TCR γδ repertoire diversity may be continually shaped by both host and microbial antigens and metabolites such that fluctuations in the frequencies of ileal γδ IELs as well as perturbations in the antimicrobial response module could lead to significant clonal revisions.

### Shared TCR clonotypes display different frequencies in ileal *Btnl2-*KO and WT γδ IELs

**Fig. 6:**
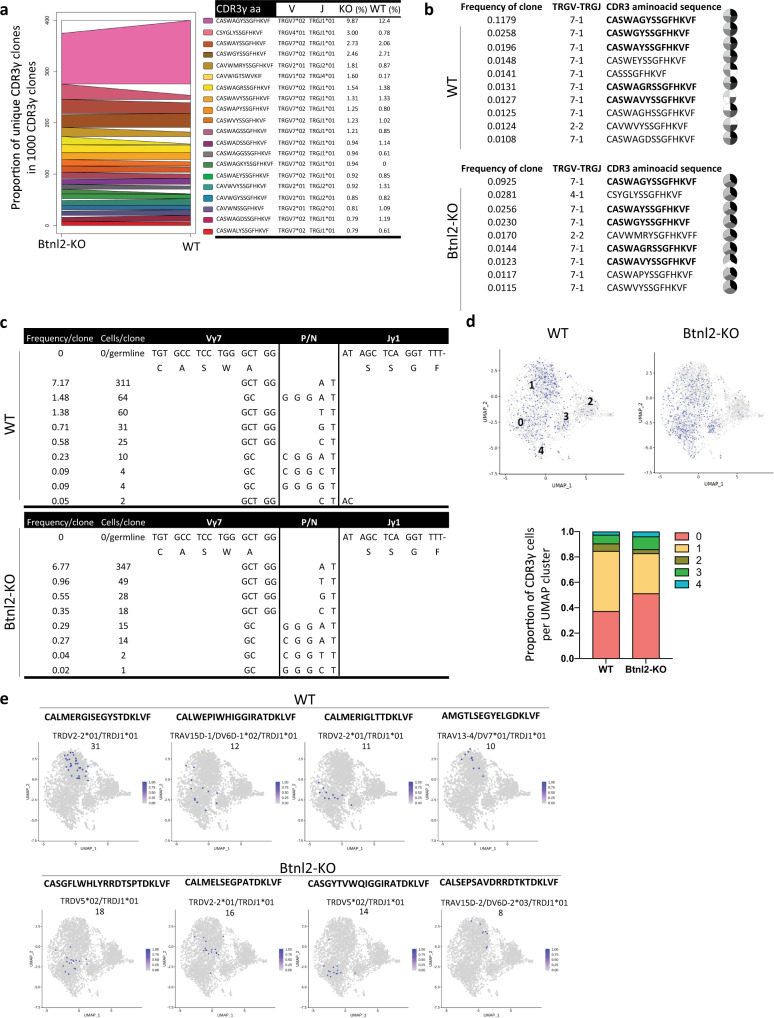
Ileal γδ IEL transcriptome of shared Btnl2-KO and WT CDR3γ clones is shaped by pairing with CDR3δ. Ileal γδ IELs from cohoused 11-week-old Btnl2-KO and WT littermates (pool of 8 mice, each) were single-cell sorted, and single-cell TCR sequencing and single-cell RNA sequencing were performed. **a** Top 20 TRG clones (CDR3γ aminoacid sequences listed in order on the right) from ileal Btnl2-KO γδ IELs, which are differentially enriched in ileal WT γδ IELs. **b** Top largest CDR3γ clones identified during scTCRseq can be found among individual ileal Btnl2-KO (*n* = 3) and ileal WT (*n* = 4) samples, in which TCR sequences have been reconstructed from bulk RNAseq. Shared clones are highlighted in bold. Each slice represents a different sample and white slices mark the absence of the CDR3γ clone from the individual sample. **c** Multiple Vγ7–J1 recombination events converge to the same top 1 CDR3γ aminoacid sequence (CASWAGYSSGFHKVF) in both ileal Btnl2-KO and ileal WT γδ IELs. **d** Distribution of top 1 CDR3γ chain (TRGV7*02/TRGJ1*01, CASWAGYSSGFHKVF; ~10% of total clones) shared by ileal Btnl2-KO and ileal WT γδ IELs among different UMAP clusters. **e** The pairing of top 1 CDR3γ (CASWAGYSSGFHKVF) with different CDR3δ sequences, listed above the UMAP plot, shapes the transcriptome of the γδ IELs. The number of clones per pair is denoted below the CDR3δ sequences.

In addition to unique *Btnl2-*KO CDR3γ clones, 19 of the top 20 ileal *Btnl2-*KO CDR3γ clones were shared by ileal WT γδ IELs as contracted or expanded clones, possibly contributing to the TRGV repertoire diversity in *Btnl2*-KO compared to WT γδ IELs (Fig. 6a**)**. Overall, ~40% of CDR3γ clones were shared by *Btnl2*-KO and WT γδ IELs in each segment (Supplementary Figure 6a, c). In TCRδ chains, each segment was characterized by a large number of unique CDR3δ clones and fewer than 3% shared clones between *Btnl2-*KO and WT mice (Supplementary Figure 6b, c). More CDR3γ and CDR3δ clones were shared by jejunal and ileal γδ IELs (131 vs. 72 and 270 vs. 179, respectively) in *Btnl2-*KO compared to WT mice, highlighting the jejunum as a transitional segment in the small intestine (Supplementary Figure 6d). *Btnl2-*KO and WT γδ IELs carrying one CDR3γ/δ pair showed virtually no overlap (less than 0.4%) of their CDR3γ/δ clonal repertoire, emphasizing that individual mice carry unique γ-chain-δ-chain pairings (Supplementary Figure 7a, b). Nevertheless, up to 13% of CDR3γ/δ paired clones overlapped between two or all three segments suggesting the presence of dominant CDR3γ/δ pairs that populate all segments within individual mice (Supplementary Figure 7c).

We next reconstructed CDR3γ sequences using bulk RNA-seq data from each individual mouse. The most frequent ileal CDR3γ clones revealed by single-cell TCR sequencing data were also found in different individual mice, which suggested that the single-cell TCR repertoire was an accurate representation of individual *Btnl2-*KO and WT CDR3γ diversities (Fig. 6b). The top ileal *Btnl2-*KO CDR3γ clones also included *TRGV4*, *TRGV1,* and *TRGV7* genes, whereas ileal WT CDR3γ clones carried *TRGV7* almost exclusively (Fig. 6c). For the most frequent CDR3γ chain (Vγ7-J1, CASWAGYSSGFHKVF), ~70% of IELs carrying this CDR3γ amino acid sequences were translated from the same DNA sequence, which could result from clonal expansion of one progenitor or from recurrent independent recombinations that pair with distict Vδ sequences in each clone, while the remainder of the IELs derived from smaller clones with different DNA sequences (Fig. 6d). Similar convergent Vγ recombination has been observed for common human Vγ9^+^ clonotypes, where their abundance has been proposed to be preconfigured since birth^48^. Likewise, the most prevalent Vγ7^+^ chain stemmed from one major and several minor independent convergent recombination events. These findings highlighted the presence of public TRGV clonotypes of γδ IELs and suggested that the paired TRGV/TRDV repertoire diversity may be driven by the CDR3δ sequence.

### γδ IEL transcriptome of shared CDR3γ clones is shaped by pairing with CDR3δ

To further explore the relationship between TCR and γδ IEL transcriptome, we performed scRNA-seq on the same duodenal, jejunal, and ileal γδ IELs we have profiled for TCR sequencing. We identified nine clusters in each sample (Supplementary Figure 8a) and the transcriptome of single γδ IELs in clusters 0, 1, and 3 clearly differentiated between duodenal and ileal origin with jejunal γδ IELs exhibiting intermediate transcriptome profiles (Supplementary Figure 8a, b).

Using the top 20 markers detected in each single cell cluster, in conjunction with molecular signatures described in recent scRNA-seq and bulk RNAseq reports^5,49^, we propose γδ IEL attributes, such as differentiation stage, maturation, and effector profile, to distinguish among γδ IEL clusters (Supplementary Figure 8c). In line with previous observations^50^, clusters 0 and 1 contain mature and highly cytolytic IELs, clusters 2 and 3 include immature IELs, whereas cluster 4 consists of newly activated IELs undergoing transcriptional changes such as antigen-mediated differentiation (Supplementary Figure 8c). The remaining IELs were subdivided into smaller clusters with specialized effector profiles such as type I/III interferon responses in cluster 5 (*Isg15*, *Irf7*, *Stat1*) and subset-specific differentiation stage such as recently emigrated CD8β^+^ IEL progenitors in cluster 6 (*Klf2*, *Thy1*, *S1pr1*, *CD8b1*, *Sell*) (Supplementary Figure 8c)^6,7,50^. With respect to *TRGV* distribution, *TRGV7* gene usage was dominant in clusters 0-4, while ileal *Btnl2*-KO γδ IELs had reduced frequencies of *TRGV7* and higher frequencies of *TRGV1* and *TRGV4* in cluster 1 compared to their WT counterparts (40.4% vs. 45.9%, 15.2% vs. 10.3%, and 13.1% vs. 9.5%, respectively; Supplementary Figure 8d).

We next examined the distribution of CDR3γ/δ pairings using the most common ileal CDR3γ, encompassing ~10% of total CDR3γ clones across different segments and genotypes (Vγ7-J1, CASWAGYSSGFHKVF). We found that the top CDR3γ chain was preferentially enriched in cluster 0 of ileal *Btnl2-*KO γδ IELs (51.3% vs. 37.2%), which is defined by the largest number of maturation and cytolytic molecules (Supplementary Figure 8c), and dominated cluster 1 in WT γδ IELs (47.4% vs 31.5%) (Fig. 6c). The pairing of the top γ chain bearing the same nucleotide sequence with distinct CDR3δ sequences shaped the transcriptome of the pairs, as they are preferentially associated with specific clusters (Fig. 6d).

Collectively, these RNA-seq and scTCR-seq observations indicate that *Btnl2* deficiency alters the transcriptome as well as the TRGV/TRDV repertoire of ileal γδ IELs, such that their antigenic specificities and antibacterial responses are changed. This report is the first to describe intestinal γδ IEL transcriptome and TCR repertoire diversity simultaneously at single-cell resolution, revealing a previously uncharacterized heterogeneity in duodenal, jejunal and ileal γδ IELs that may account for compartment-specific immune responses driven by tissue-specific expression of immune-modulatory molecules.

### *Btnl2-*KO mice exhibit more severe intestinal inflammation in chronic DSS-induced colitis

**Fig. 7:**
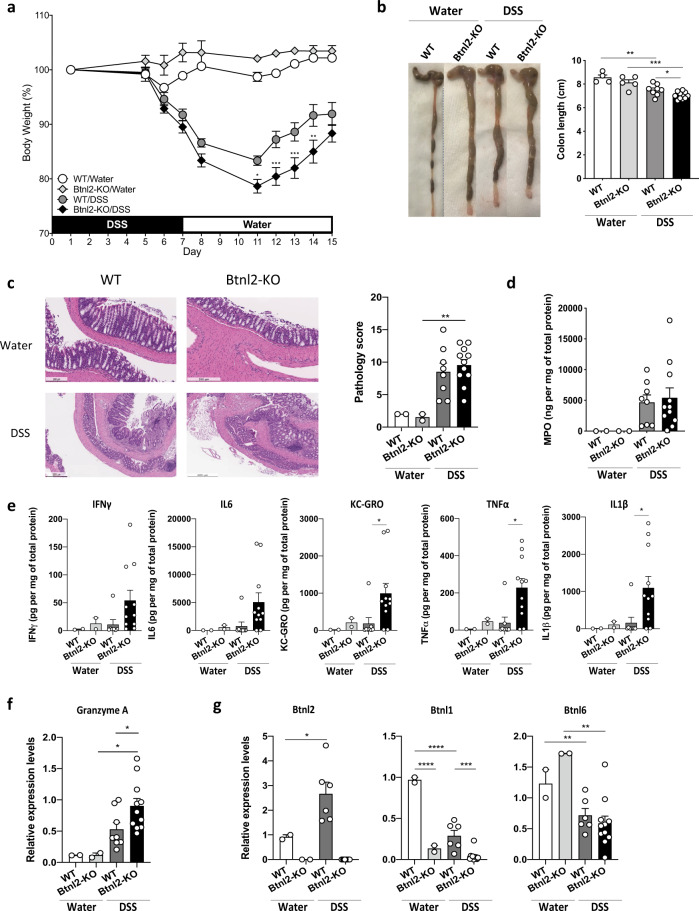
Btnl2-KO mice exhibit more severe intestinal inflammation in chronic DSS-induced colitis. Cohoused 15-week-old Btnl2-KO (*n* = 11) and WT (*n* = 8) littermates were subjected to 3% DSS-induced colitis for 7 days followed by water for 8 days. Control mice (*n* = 2–4) received water. **a** Body weight loss in cohoused Btnl2-KO and WT littermates calculated as the percent difference between the initial and actual body weight on the above days. Error bars represent mean ± SEM. Significance is measured using unpaired *t*-tests assuming similar SD, *p < 0.05, **p < 0.005, ***p < 0.0005, significantly different from DSS-treated WT mice. **b** Colon length of water- and DSS-treated Btnl2-KO and WT mice on day 15. **c** H&E histological sections and a pathological score of the colon from water- and DSS-treated Btnl2-KO and WT mice. Scale bars are 200 μm (WT/water), 250 μm (Btnl2-KO/water), 500 μm (WT/DSS and Btnl2-KO/DSS). **d** Myeloperoxidase (MPO) activity in colon homogenates of water- and DSS-treated Btnl2-KO and WT mice. **e** Levels of pro-inflammatory cytokines in colon homogenates of water and DSS-treated Btnl2-KO and WT mice. **f** Granzyme A mRNA levels in colon homogenates of water- and DSS-treated Btnl2-KO and WT mice, normalized to β2m. Error bars represent mean ± SEM. Significance is measured using unpaired t-tests assuming similar SD, *p < 0.05. **g** Btnl1/2/6 mRNA levels in the colon of water- and DSS-treated WT mice, normalized to β2m. Error bars represent mean ± SEM. Significance is measured using one-way ANOVA, *p < 0.05, **p < 0.005, ***p < 0.0005.

Since *BTNL2* SNPs have been associated with increased risk of UC and Crohn’s disease (CD)^31,51–53^, we assessed the impact of its deficiency on mucosal immune responses in the setting of DSS-induced epithelial injury^54,55^. Briefly, cohoused *Btnl2-*KO and WT littermates were subjected to DSS-induced colitis by administering DSS for 7 days followed by 8 days of water. While *Btnl2-*KO and WT mice exhibited comparable intestinal damage in the early phase of the disease (day 7), as demonstrated by comparable body weight loss and increased myeloperoxidase activity (MPO) levels, a biomarker of intestinal injury and neutrophilia^56^ (Fig. 7a, d), we observed that *Btnl2-*KO mice exhibited a significant delay in body weight recovery compared to WT littermates during the repair phase of colitis (Fig. 7a). The observed delay in recovery was accompanied by significantly shorter colons, increased granzyme A levels, greater histopathological damage, and MPO activity in the colon compared to WT littermates (Fig. 7b–d, f). Notably, DSS-treated *Btnl2*-KO mice had ~2-fold higher levels of pro-inflammatory cytokines such as IFNγ, IL-6, KC-GRO, TNFα, and IL-1β in the colon (Fig. 7e). In contrast, MPO activity, a pro-inflammatory cytokine, and granzyme A levels were not significantly altered in the ileum of DSS-treated *Btnl2-*KO mice compared to WT littermates (Supplementary Figure 9a–c). Corroborating these results, *Btnl2* transcripts were increased in the colon of DSS-treated WT mice, whereas the levels of other family members, such as *Btnl1* and *Btnl6*, were decreased with DSS treatment (Fig. 7g). *Btnl2* transcripts were unchanged in the ileum of DSS-treated suggesting that *Btnl2* expression in the colon may be induced as a feedback regulatory mechanism at the site of injury to attenuate DSS-triggered inflammation and facilitate the recovery process (Supplementary Figure 9d).

## Discussion

Emerging research places the Btn/Btnl family of molecules at the heart of γδ T cell development. Our studies shed light on Btnl2 as a regulator of ileal γδ IEL maintenance. Specifically, we propose that Btnl2 acts as a *coinhibitory* ligand to an unidentified receptor(s) on γδ IELs and regulates both proliferation and segment-specific effector profiles of ileal γδ IELs under homeostatic conditions.

Through our segment-focused approach, we found a temporal and spatial window during which Btnl2 exerted its functions on intestinal γδ IELs. ScTCRseq revealed that Vγ7^+^ IELs dominated the small intestine of 11-week-old *Btnl2-*KO and WT mice suggesting that their development was not affected. Although Btnl2 impacted γδ IEL proliferation preferentially in the ileum, its deficiency reverberated throughout distinct segments of the small intestine. Specifically, despite the ability of Btnl2 to suppress both duodenal and jejunal/ileal γδ IEL proliferation in vitro, this was confined in vivo only to ileal γδ IEL expansion. However, at the molecular level, *Btnl2* deficiency led to an altered Vγ usage among Vγ7^−^ IELs and similarly altered Vδ usage across all three segments of the small intestine suggesting overlapping as well as unique roles for Btnl2 across the distinct segments of the small intestine. This in turn was accompanied by dysregulated antibacterial module in ileal *Btnl2-*KO γδ IELs, which may be relevant for mucosal repair and clearance of segment-tropic pathogenic microbes^9^.

Consistent with a region-specific effect, duodenal *Btnl2-*KO CDR3γ and CDR3γ/δ clonal repertoires were not markedly different from those identified in cohoused WT littermates underscoring the ileum as the predominant site of Btnl2-mediated regulation at steady-state. Importantly, most abundant Vγ clones in the ileal compartment of individual *Btnl2-*KO mice included Vγ1^+^ and Vγ4^+^ clones, in contrast to Vγ7^+^ clones exclusively enriched in WT mice. *Btnl2* may be important for co-regulating ligands (i.e. Btnl1, Btnl6) of Vγ7^+^ TCRs during early adulthood. In support of this dynamic remodeling of the γδ TCRs, *Btnl2* deficiency led to different convergent recombination events, such that pairing of the most common Vγ7^+^ chain with distinct Vδ sequences defined the transcriptome profiles of ileal γδ IELs. Based on the previous studies^5,17^, one possibility is that site-specific metabolite levels and/or antigenic pressure led to multiple independent in situ recombination events, suggesting an adaptive behavior of γδ IELs towards local environmental antigens. Since ileal γδ IEL motility along the villi-crypt axis is strictly dependent on the presence of microbiota^14^, an impaired antibacterial profile could also be a consequence of improper localization or ineffective surveillance of ileal *Btnl2-*KO γδ IELs. Conversely, loss of epithelia-expressed Btnl2 could lead to alterations in the local microbiome, which would then drive reshaping of the TCR repertoire and antibacterial response module of ileal *Btnl2-*KO γδ IELs. Further studies are required to understand how the reshaped Vγ-Vδ repertoire alongside the defective antibacterial response module may affect the susceptibility of *Btnl2-*KO mice to small intestinal infectious agents.

An acute reliance on Btnl expression at a predefined time has been proposed for both murine Vγ7^+^ IEL development and human Vγ4^+^/Vδ1^+^ IEL maintenance^5,7^. Specifically, Btnl1 expression in adult *Btnl1*-KO mice could not rescue Vγ7^+^ development^7^, whereas mucosal repair and Btnl8 expression restoration following adherence to a gluten-free diet could not reconstitute Vγ4^+^/Vδ1^+^ IEL subsets in patients with celiac disease^5^. In light of these observations, it is tempting to speculate that Btnl molecules may regulate not only the selective expansion of tissue-specific Vγ chains in neonates but also their TCR specificities across distinct tissue compartments in young adults. As such, segment-biased γδ TCR specificities may be determined by the choice of dimerization partners among Btnl molecules and their nuanced spatial and temporal expression in the intestine. While Btnl1 and Btnl6 jointly affect Vγ7 selection and maturation, there is no known binding partner for Btnl2. In addition, no other Btnl molecules were induced in the intestine to compensate for the loss of Btnl2 suggesting that their expression patterns were not co-regulated, despite being encoded at the same locus. Of the various family members, structurally, Btnl2 is unique in that it lacks the antigen-binding B30.2 domain shared by most of the Btn/Btnl superfamily members^57^, suggesting that the inhibitory effect of Btnl2 may depend on the signaling pathways triggered downstream of engagement of its putative receptor on γδ IELs. Btnl2 could either homodimerize or heterodimerize with other intestine-specific Btnls through IgC interactions independent of B30.2 domains^42,58^. As a heterodimer, Btnl2 interacting partner may contribute to the B30.2-driven activation of the heterodimer and binding to the putative receptor, whereby Btnl2 ligation would induce the downstream inhibition of proliferation. Btnl1, Btnl4, and Btnl6 can be candidate binding partners of Btnl2 due to their similar intestinal expression^7^. Hence, despite higher expression on duodenal IECs, Btnl2 may exert more profound inhibition on ileal γδ IELs due to increased regional expression of its binding partner on IECs and putative receptor on γδ IELs. As such, this region-specific interaction may be promoted by local soluble antigens like bacterial metabolites. Btnl2 could also function as a receptor antagonist prohibiting the binding of another Btnl heterodimer to γδ TCR and suppressing γδ IEL proliferation. Alternatively, as this suppression is only partial, Btnl2 may indirectly target certain Vγ TCR(s) or TCR specificities via regulating surface expression of Vγ ligands (i.e. Btnl6 for Vγ7 TCRs)^16,59^. Further studies are required to address whether Btnl2 can exert its inhibitory effects on γδ IELs across all intestinal compartments during segment-specific inflammation.

Previous studies showed that γδ T cell depletion induces greater colonic damage, reduced KGF secretion, increased IFNγ production by αβ T cells and decreased IEC proliferation during DSS-induced colitis, suggesting that γδ IELs can promote mucosal repair following epithelial injury^12,60^. Conversely, impaired IL-10 production by Tregs leads to uncontrolled γδ IEL proliferation and spontaneous colitis in *Pdk1*^*f/f*^*; CD4*^*cre*^ mice, supporting a proinflammatory role for γδ IELs in the colon^61^. *Btnl2* expression is upregulated in the distal colon during DSS-induced colitis and *Btnl2-*KO colitic mice exhibit a delay in recovery during the mucosal repair phase of the disease, potentially due to γδ IEL-dependent and -independent (i.e. Tregs, proinflammatory helper T cells) mechanisms to controlling the damage caused by epithelial injury. Interestingly, Btnl1 and Btnl6 transcripts were downregulated in *Btnl2-*KO colitic mice, confirming previous reports in which Btnl1/6 transcripts were shown to be significantly reduced in the distal colon of *Muc2*-KO mice and BTNL8 expression was diminished or lost in colonic and duodenal biopsies of patients with UC and celiac disease, respectively^5,32^. Hence, we speculate that IECs upregulate Btnl2 expression in response to environmental stress factors to limit the damage-induced expansion of γδ IELs and induce the release of antibacterial molecules. As such, our findings support the idea that close interaction between γδ IELs and epithelium-specific Btnl molecules throughout the small and large intestines drives their proliferation and function in homeostatic and inflammatory settings^14^.

In conclusion, we have unveiled a novel role for Btnl2 in regulating the expansion of ileal γδ IELs, sculpting of their Vγ and Vγ-Vδ TCR specificities and altering their antibacterial response module. Our scRNAseq and scTCRseq surveys revealed a highly dynamic γδ IEL compartment finely adapted to environmental cues of each segment of the small intestine during adulthood. Further studies are required to establish whether the timing and choice of intestinal Btnl heteromers drive the site-specific functions of γδ IELs in intestinal immune disorders. Taken together, these studies suggest that Btnl-mediated targeting of γδ IEL development and maintenance may help dissect their immunological functions in intestinal diseases with gut segment-specific manifestations.

## Materials and methods

### Mice

Eight- to twelve-week-old female C57BL/6 mice were obtained from Jackson Laboratory. *Btnl2-*KO mice on a C57BL/6 background were generated and maintained at Regeneron Pharmaceuticals Inc. using the VelociGene technology^62,63^. Briefly, a *LacZ* cassette was inserted in-frame with the start codon followed by a selection cassette that disrupted the transcription of the *Btnl2* gene resulting in a null allele. Heterozygous mice were interbred to produce homozygous KO and WT littermates. *Btnl2* expression pattern was confirmed by β-galactosidase staining and *Btnl2* targeted deletion was measured by quantitative RT-PCR and RNA sequencing of the small intestine. *Btnl2*-KO and WT female mice were used at 10-17 weeks of age for all the experiments except when otherwise indicated. Female littermates were cohoused after weaning for several weeks and assigned randomly to experimental groups in disease settings. All animals were maintained under pathogen-free conditions and experiments were performed according to protocols approved by the Institutional Animal Care and Use Committee at Regeneron Pharmaceuticals Inc.

### Isolation of intestinal epithelial cells (IECs), intraepithelial lymphocytes (IELs), and lamina propria lymphocytes (LPLs)

The small intestine was divided into three equal segments and lymphocyte isolation proceeded as described previously^64^. Briefly, to isolate IEC and IEL fractions, the small intestine was cut into 2 cm pieces and incubated in HBSS containing 5 mM EDTA, 10 mM HEPES and 2% fetal calf serum (FCS) twice for 15 min at 37 °C with shaking at 150 rpm. After vigorous vortexing, the intestinal pieces were washed over 100 μm cell strainer and centrifuged on a 40%/80% Percoll gradient (GE Healthcare) at 2500 rpm for 20 min at 20 °C. The top layer containing IECs was collected, washed, and resuspended in Trizol for RNA extraction. IEL fraction was collected from the interface, washed, and resuspended in Miltenyi MACS buffer. Following IEL isolation, LPLs were isolated from intestinal pieces by incubation in HBSS w/o Ca^2+^/Mg^2+^ supplemented with 50 U mL^−1^ Collagenase D (Roche), 0.25 mg mL^−1^ DNase I (Sigma-Aldrich), 50 U mL^−1^ Dispase (Corning), and 5% FCS for two rounds of 25 min at 37 °C with shaking at 150 rpm. Cells were centrifuged on a 40%/80% Percoll gradient (GE Healthcare) and LPLs were collected from the interface, washed, and resuspended in MACS buffer for immediate surface cell staining.

### Mesenteric lymph node and Peyer’s Patch immunophenotyping

Peyer’s Patches were collected from the whole small intestine, washed with ice-cold DPBS, and incubated with 50 U mL^−1^ Collagenase D (Roche), 0.25 mg mL^−1^ DNase I (Sigma-Aldrich), 50 U mL^−1^ Dispase (Corning), and 5% FCS for 25 min at 37 °C with shaking at 150 rpm. Mesenteric lymph nodes were minced in HBSS with Ca^2+^/Mg^2+^containing 15 U mL^−1^ Collagenase D (Roche) and 50 μg mL^−1^ DNase I (Sigma-Aldrich), and incubated for 20 min at 37 °C without shaking. Cells were resuspended in MACS buffer for immediate surface staining.

### Flow cytometry

Flow cytometry antibodies were purchased from Biolegend (US), BD Biosciences (US), TONBO Biosciences (US), eBioscience (US) and ThermoFischer (US). Dead cells were excluded using LIVE/DEAD fixable blue dead cell stain (Thermo Fischer Scientific, Cat#L23105). Fc receptors were blocked using purified anti-mouse CD16/32 (BD Pharmigen, Clone 2.4G2, Cat#553142) and 2% each of normal mouse serum (Jackson ImmunoResearch, Cat#015-000-120), rat serum (Jackson ImmunoResearch, Cat#012-000-120) and hamster serum (Jackson ImmunoResearch, Cat#007-000-120). The following antibodies were used for the staining according to manufacturer’s instructions: CD45-BV510 (Biolegend, Clone#30-F11, Cat#103138), CD8α-AF700 (Biolegend, Clone#53-6.7, Cat#100730), CD8β-PerCP/Cy5.5 (Biolegend, Clone#YTS156.7.7, Cat#126610), TCRβ-BV711 (Biolegend, Clone#H57-597, Cat#109243), TCRβ-APC/Cy7 (Biolegend, Clone#H57-597, Cat#109220), TCRγ/δ-PE/Cy7 (Biolegend, Clone#GL3, Cat#118124), CD11b-APC/Cy7 (Biolegend, Clone#M1/70, Cat#101226), CD11c-APC/Cy7 (Biolegend, Clone#N418, Cat#117324), CD11c-AF700 (Biolegend, Clone#N418, Cat#117320), CD11c-PE/Cy7 (Biolegend, Clone#N418, Cat#117318), Gr1-APC/Cy7 (Biolegend, Clone#RB6-8C5, Cat#108424), B220-APC/Cy7 (Biolegend, Clone#RA3-6B2, Cat#103224), B220-AF700 (Biolegend, Clone#RA3-6B2, Cat#103232), B220-BV650 (Biolegend, Clone#RA3-6B2, Cat#103241), NK1.1-APC/Cy7 (Biolegend, Clone#PK136, Cat#108724), MHCII-BV421 (Biolegend, Clone#M5/114.15.2, Cat#107632), Ly6C-PerCP/Cy5.5 (Biolegend, Clone#HK1.4, Cat#128012), CD64-PE (Biolegend, Clone#X54-5/7.1, Cat#139304), CD103-FITC (Biolegend, Clone#2E7, Cat#121420), CX3CR1-Biotin (Biolegend, Clone#SA011F11, Cat#149018), Streptavidin-PE/Dazzle 594 (Biolegend, Cat#405248), NKp46-PE/Dazzle594 (Biolegend, Clone#29A1.4, Cat#137630), CD4-PerCP/Cy5.5 (Biolegend, Clone#GK1.5, Cat#100434), CD4-VF450 (Tonbo Biosciences, Clone#GK1.5, Cat#75-0041-U100), c-KIT-PE-Cy7 (Biolegend, Clone#ACK2, Cat#135112), CD44-APC/Cy7 (Biolegend, Clone#IM7, Cat#103028), CD44-BV650 (Biolegend, Clone#IM7, Cat#103049), RORγt-APC (eBiosciences, Clone#AFKJS-9, Cat#17-6988-82), FoxP3-AF700 (eBioscience, Clone#FJK-16s, Cat#56-5773-82), FoxP3-eF450 (eBioscience, Clone#FJK-16s, Cat#48-5773-82), LegendScreen Mouse PE kit (Biolegend, Cat#700005). For intranuclear staining, cells were incubated with fixable viability dye and surface markers prior to fixation and permeabilization using the FoxP3/Transcription factor fixation and permeabilization kit (eBioscience) according to manufacturer’s instructions.

BrdU (Sigma-Aldrich) incorporation was assessed 3 days after continuous administration in drinking water dissolved at 0.8 mg mL^−1^ in 3% sucrose. Briefly, IELs were fixed with BD Cytofix/Cytoperm™ for 20 min at 20 °C, washed and incubated with 1x DPBS supplemented with Ca^2+^/Mg^2+^, 10% FCS, and 10% DMSO for 10 min at 20 °C. Cells were re-fixed with BD Cytofix/Cytoperm™ for 5 min at 20 °C, washed and incubated with 0.5 mg mL^−1^ DNAse I (Sigma-Aldrich) for 1 hr at 37 °C. Cells were stained with BrdU-AF647 (MoBU-1) (Thermo Fisher Scientific, Clone# MoBU-1, Cat#B35133) at 20 °C, washed, and resuspended for acquisition.

Flow cytometry was performed on the LSRFortessa X-20 instrument (BD Biosciences), data were analyzed using FlowJo software (BD Biosciences) and plotted using GraphPad Prism™ (GraphPad Software, Inc.). Representative gating strategies for flow cytometry are provided in Supplementary Figure 10.

### In vitro IEL proliferation assay

Total 96-well flat-bottom plates were coated overnight with 1 μg mL^−1^ purified anti-mouse CD3ε (Tonbo Biosciences, 145-2C11, Cat#70-0031-M001) and 60 pmoles of mouse Btnl2-Fc, PDL1-Fc or mFc (Adipogen Life Sciences) at 4 °C and washed twice with DPBS before adding IELs to the cultures. Freshly isolated IELs were labeled with CellTrace CFSE Cell Proliferation dye according to the manufacturer’s instructions (Thermo Fischer Scientific, Cat#C34554). CFSE-labeled IELs were plated at 200,000 cells per well in RPMI 1640 supplemented with 10% FCS, 1% Pen/Strep, 2% HEPES, 1% Glutamine, 1% nonessential amino acids, 1% sodium pyruvate, 0.1% β-mercapto-ethanol (Gibco), recombinant mouse IL-7 (10 ng mL^−1^, R&D), recombinant mouse IL-15 (10 ng mL^−1^, R&D) and recombinant human IL-2 (10 ng mL^−1^, Peprotech). Cells were incubated for 72–96 h at 37 °C in 5% CO_2_ prior to analysis.

### In vitro CD4^+^ T cell proliferation assay

Total 96-well flat-bottom plates were coated overnight with 1 μg mL^−1^ purified anti-mouse CD3ε (Tonbo Biosciences, 145-2C11, Cat#70-0031-M001), 1 μg mL^−1^ purified anti-mouse CD28 (Tonbo Biosciences, 37.51, Cat#70-0281-U500), and 60 pmoles of mouse Btnl2-Fc, PDL1-Fc, PDL2-Fc or mFc (Adipogen Life Sciences) at 4 °C and washed twice with DPBS before adding CD4^+^ T cells to the cultures. CD4^+^ T cells were enriched from pooled spleen and lymph nodes using mouse CD4 (L3T4) microbeads (Miltenyi Biotec) and labeled with CellTrace CFSE Cell Proliferation dye (Thermo Fischer Scientific, Cat#C34554). CFSE-labeled CD4^+^ T cells were plated at 80,000–100,000 cells per well in RPMI 1640 supplemented with 10% FCS, Pen/Strep, 2% HEPES, 1% Glutamine, 1% nonessential amino acids, 1% sodium pyruvate and 0.1% β-mercapto-ethanol (Gibco). Cells were incubated for 72 h at 37 °C in 5% CO_2_ prior to analysis.

### DSS-induced model of colitis

Fourteen-twenty-week-old cohoused female *Btnl2-*KO and WT mice with an average body weight greater than 23 g were given 3% DSS (Sigma-Aldrich) in drinking water for 6–7 days followed by distilled water for up to 10 days. Control group received only distilled water for the duration of the study. Mice were weighed and monitored daily for clinical signs of colitis (e.g. stool consistency, fecal blood). On day 15, mice were euthanized, and colon length was measured.

### Generation of colon and ileal homogenates and measurement of cytokines and myeloperoxidase (MPO) activity

Total 6 mm pieces of distal colon or terminal ileum were placed in T-per buffer (Thermo Fisher Scientific) containing 1× Halt Protease Inhibitor Cocktail (Thermo Fisher Scientific), 0.5 M EDTA solution (Thermo Fisher Scientific), and two 3 mm tungsten carbide beads (Qiagen). Tissues were disrupted in TissueLyser II (Qiagen) for 10 min at an oscillation frequency of 27.5 Hz. Generated tissue homogenates were centrifuged at 15000 rcf for 10 min at 4 °C and the supernatants were collected into deep 96-well plates. Protein assay dye (BioRad) was used to quantify total protein content using Bradford protein assay according to the manufacturer’s instructions. Cytokine concentrations were measured using V-PLEX Plus Proinflammatory Panel 1 mouse kit according to the manufacturer’s instructions (Meso Scale Diagnostics). Absorbance was measured on the Meso SECTOR S600 instrument (Meso Scale Diagnostics). Myeloperoxidase (MPO) activity was measured using a mouse MPO ELISA kit according to the manufacturer’s instructions (Hycult Biotech). Absorbance was measured on the SpectraMax i3x instrument (Molecular Devices). Data analysis was performed using GraphPad Prism™ (GraphPad Software, Inc.). Cytokine and MPO levels were normalized to total protein content.

### Histology

Total 3 cm pieces of duodenum, jejunum, ileum, and colon were prepared as swiss rolls, fixed in 10% buffered formalin, embedded in paraffin, sectioned at 5 μm and H&E stained. Histology was performed by HistoWiz Inc. (histowiz.com) using a Standard Operating Procedure and fully automated workflow. After staining, sections were dehydrated and film coverslipped using a TissueTek-Prisma and Coverslipper (Sakura). Whole slide scanning (40x) was performed on an Aperio AT2 (Leica Biosystems). Histopathological scoring was performed by an evaluator blinded to genotype, group assignment, and experimental outcome. The following features were evaluated for DSS-induced injury and scored based on previous published criteria:^65^ degree of inflammation in lamina propria, goblet cell loss, abnormal crypts, presence of crypt abscesses, mucosal erosion, and ulceration, submucosal spread to transmural involvement, number of neutrophils. Each parameter received a score from 0 to 4 with a maximum cumulative score of 17. Mucosal lesions in unchallenged mice were scored as described previously:^66^ 0, normal; 1, mild sloughing of epithelial cells; 2, moderate sloughing of epithelial cells; 3, severe mucosal edema; 4, extensive mucosal injury. Data analysis was performed using GraphPad Prism™.

### Quantitative PCR

RNA was isolated from IECs derived from duodenum, jejunum, ileum, and colon from cohoused unchallenged *Btnl2-*KO and WT mice. RNA was extracted from distal colon and terminal ileum from cohoused, water- and DSS-treated *Btnl2-*KO and WT mice. RNA was purified on Kingfisher flex (Thermo Fisher Scientific) using the MagMAX-96 for Microarrays Total RNA isolation kit (Thermo Fisher Scientific) with an additional DNAse I (Sigma-Aldrich) step added between the first and second washes. cDNA synthesis was performed using SuperScript® VILO™ Master mix (Thermo Fisher Scientific) according to the manufacturer’s instructions. qPCR was performed using MyTaq™ Mix (Bioline) and assay mix (Thermo Fisher Scientific or LGC BioSearch). Probes for each gene are listed in Table 1. qPCR was run on an ABI 7900HT Fast Real-Time PCR System with a 384-well block module and automation accessory (Thermo Fisher Scientific). Gene expression was normalized to β2 m and differences were determined using the 2ΔC(t) calculation.

**Table 1:** RT-PCR Probes for select genes.

Gene	Forward	Reverse	Probe
BTNL2	GGATTGCCCACGGTATAGTC	AGGACCGACCACTCTGAAG	TATCTGGCGTGGCTGCCTCCTT
BTNL1	GGTGCAGATGCCGGAATACAG	GCCACACTTCCCATGTCAATG	CAGGACCCAGATGGTGAGACAAGC
BTNL6	GAGGCCATCTTGGAACTGAA	CCACCGTCTTCTGGACCTTT	TGGCAGCAATGGGCTCTGTCC
b2m	GGGAAGCCGAACATACTGAACTG	CCCGTTCTTCAGCATTTGGATTTC	ACGTAACACAGTTCCACCCGCCT
Gzma	GGCGCTTTGATTGAAAAGAACTG	TGTTCTGGCTCCTTATTGATTGAG	TGACTGCTGCCCACTGTAACGTGG

### Single-cell RNA sequencing of γδ IELs

Following IEL isolation, cells were stained with LIVE/DEAD fixable blue dead cell stain as per the manufacturer’s instructions (Thermo Fisher Scientific, Cat#L23105). Fc receptors were blocked using purified anti-mouse CD16/32 (BD Pharmigen, Clone 2.4G2, Cat#553142) and 2% each of normal mouse serum (Jackson ImmunoResearch, Cat#015-000-120), rat serum (Jackson ImmunoResearch, Cat#012-000-120) and hamster serum (Jackson ImmunoResearch, Cat#007-000-120) and cells were stained using the following antibodies: CD45-BV510 (Biolegend, Clone#30-F11, Cat#103138), CD8α-AF700 (Biolegend, Clone#53-6.7, Cat#100730), CD8β-PerCP/Cy5.5 (Biolegend, Clone#YTS156.7.7, Cat#126610), TCRβ-BV711 (Biolegend, Clone#H57-597, Cat#109243), TCRγδ-PE/Cy7 (Biolegend, Clone#GL3, Cat#118124). Two mice were pooled per each sample. γδ IELs were sorted from each sample using MoFlo Astrios EQ (Beckman Coulter). Two-thirds of each sample were resuspended in RNA Lysis Buffer (Zymo Research) and processed for bulk RNA sequencing. One-third of each sample was pooled per segment of the small intestine per genotype, resuspended in PBS with 0.04% BSA, and loaded on a Chromium Single Cell Instrument (10X Genomics). RNAseq and V(D)J libraries were prepared using Chromium Single Cell 5’ Library, Gel Beads & Multiplex Kit (10X Genomics). After amplification, cDNA was divided into RNAseq and V(D)J library aliquots. To enrich the V(D)J library aliquot for γδ TCRs, cDNA was divided into two 10 ng aliquots and amplified in two rounds using internally designed primers. In particular, the following primers were used for the first round of amplification: MP147 for short R1 (ACACTCTTTCCCTACACGACGC), MP371 for mouse TRGC1-3 (/5Biosg/TTCCTGGGAGTCCAGGATRGTATTG), MP 372 for mouse TRGC4 (/5Biosg/CACCCTTATGACTTCAGGAAAGAACTTT), and MP369 for mouse TRDC (/5Biosg/TTCCACAATCTTCTTGGATGATCTGAG). For the second round of amplification, 20 ng aliquots from the first round were further amplified using MP147 for short R1 (ACACTCTTTCCCTACACGACGC), MP373 a nested R2 plus mouse TRGC(GTGACTGGAGTTCAGACGTGTGCTCTTCCGATCTGTCCCAGYCTTATGGAGATTTGT), and MP370 a nested R2 plus mouse TRDC (GTGACTGGAGTTCAGACGTGTGCTCTTCCGATCTTAGTCACCTCTTTAGGGTAGAAATCTT). V(D)J libraries were prepared from 25 ng of each mTRGC and mTRDC amplified cDNA. Paired-end sequencing was performed on Illumina NextSeq500 for RNAseq libraries (Read 1 26 bp for a unique molecular identifier (UMI) and cell barcode, 8 bp i7 sample index, 0 bp i5, and Read 2 55 bp transcript read) and V(D)J libraries (Read 1 150 bp, 8 bp i7 sample index, 0 bp i5, and Read 2 150 bp read). For RNAseq libraries, Cell Ranger Single-Cell Software Suite (10X Genomics, v2.2.0) was used to perform sample de-multiplexing, alignment, filtering, and UMI counting. The mouse mm 10 genome assembly and RefSeq gene model for mouse were used for the alignment. For V(D)J libraries, Cell Ranger Single-Cell Software Suite (10X Genomics, v2.2.0) was used to perform sample de-multiplexing, de novo assembly of read pairs into contigs, align and annotate contigs against all the germline segment V(D)J reference sequences from IMGT, label and locate CDR3 regions, group clonotypes.

### Single-cell RNA sequencing data analysis

scRNAseq data were analyzed using Seurat R package^67^. Cells with fewer than 500 genes or more than 10% of mitochondrial RNA content were excluded during the quality control (QC) step. The remaining cells underwent dimension reduction by PCA on the highly variable genes. Data were further reduced to the 2D space on the first 20 PCs using uniform manifold approximation and projection (UMAP). Cell clusters were determined using a graph-based unbiased clustering approach implemented in Seurat. Positive markers defining each cluster were identified using the Wilcoxon rank-sum test. Six representative markers were selected for each cluster to visualize in heatmaps.

### Single-cell TCR sequencing data analysis

After V(D)J sequences were assembled and annotated, only productive γ and δ TCR sequences were kept. Two TCR diversity metrics (i.e. species richness and exponential of Shannon entropy) were estimated for each sample using iNEXT R package^47,68^. Species richness measured total unique clone numbers, whereas the Shannon index computed the uncertainty in predicting the identity of a sequence taken at random from the dataset. Both interpolated and extrapolated diversities were estimated, and a 95% confidence interval was based on 50 bootstraps. TCR repertoires were visualized using the Treemap R package (https://CRAN.R-project.org/package=treemap). Downstream TCR analysis such as V(D)J usage, shared TCR, and integration of TCR and RNA-seq was performed using customized R scripts (available upon request).

### Bulk RNA-sequencing and data analysis

cDNA was synthesized and amplified (16-cycle PCR) from 5 ng total RNA using SMARTer® Ultra® Low RNA Kit (Clontech). Nextera XT library prep kit (Illumina) was used to generate the final sequencing library (12 PCR cycles performed to amplify libraries) using 1 ng of cDNA as the input. The amplified libraries were size-selected at 400 to 600 bp. Sequencing was performed on Illumina HiSeq® 2500 (Illumina) by multiplexed paired-read run with 2 × 100 cycles. The sequencing reads were mapped to the customized mouse genome using ArrayStudio (OmicSoft). Sense-strand exon reads were used to quantify the gene expression level by RSEM algorithm implemented in ArrayStudio. Genes were flagged as detectable with a minimum of 10 reads. Differentially expressed gene analysis was performed using Deseq2^69^. Genes with fold change | > 1.5 | and FDR < 0.05 were considered significantly differentially expressed. The differentially expressed genes were subjected to pathway enrichment analysis using the Running Fish exact test in NextBio (www.nextbio.com). TCR hypervariable-region sequences were reconstructed using TRUST^70^.

### Statistics and reproducibility

Statistical significance (*p* values) within the groups was determined by using one of the following statistical tests: unpaired *t*-tests assuming similar SD; one-way ANOVA with Tukey’s multiple comparison post-test; or ordinary two-way ANOVA with Sidak’s multiple comparison post-test, *p < 0.05, **p < 0.005, ***p < 0.0005. *P* values of < 0.05 were considered significant. Statistical analyses were performed with Graphpad Prism 8. Samples were defined as biological replicates and no technical replicates were used to generate graphs. Each experiment was repeated at least three times, sample sizes and numbers and the statistical test used were indicated in each figure legend.

### Reporting summary

Further information on research design is available in the Nature Research Reporting Summary linked to this article.

## Supplementary information

Supplementary Information

Description of Additional Supplementary Files

Supplementary Data 1

Reporting Summary

## Data Availability

The single-cell RNA-seq, TCR seq, and bulk RNA-seq data have been deposited to Gene Expression Omnibus under accession number (GSE178273). Source data can be found in Supplementary Data 1.
