# Supplementary material for: Butyrophilin-like 2 regulates site-specific adaptations of intestinal γδ intraepithelial lymphocytes
Source: Commun Biol. 2021 Jul 26;4:913. doi: 10.1038/s42003-021-02438-x (PMC8313535; doi:10.1038/s42003-021-02438-x)
Supplement: Supplementary file 1 — Supplementary Information [file 42003_2021_2438_MOESM1_ESM.pdf]

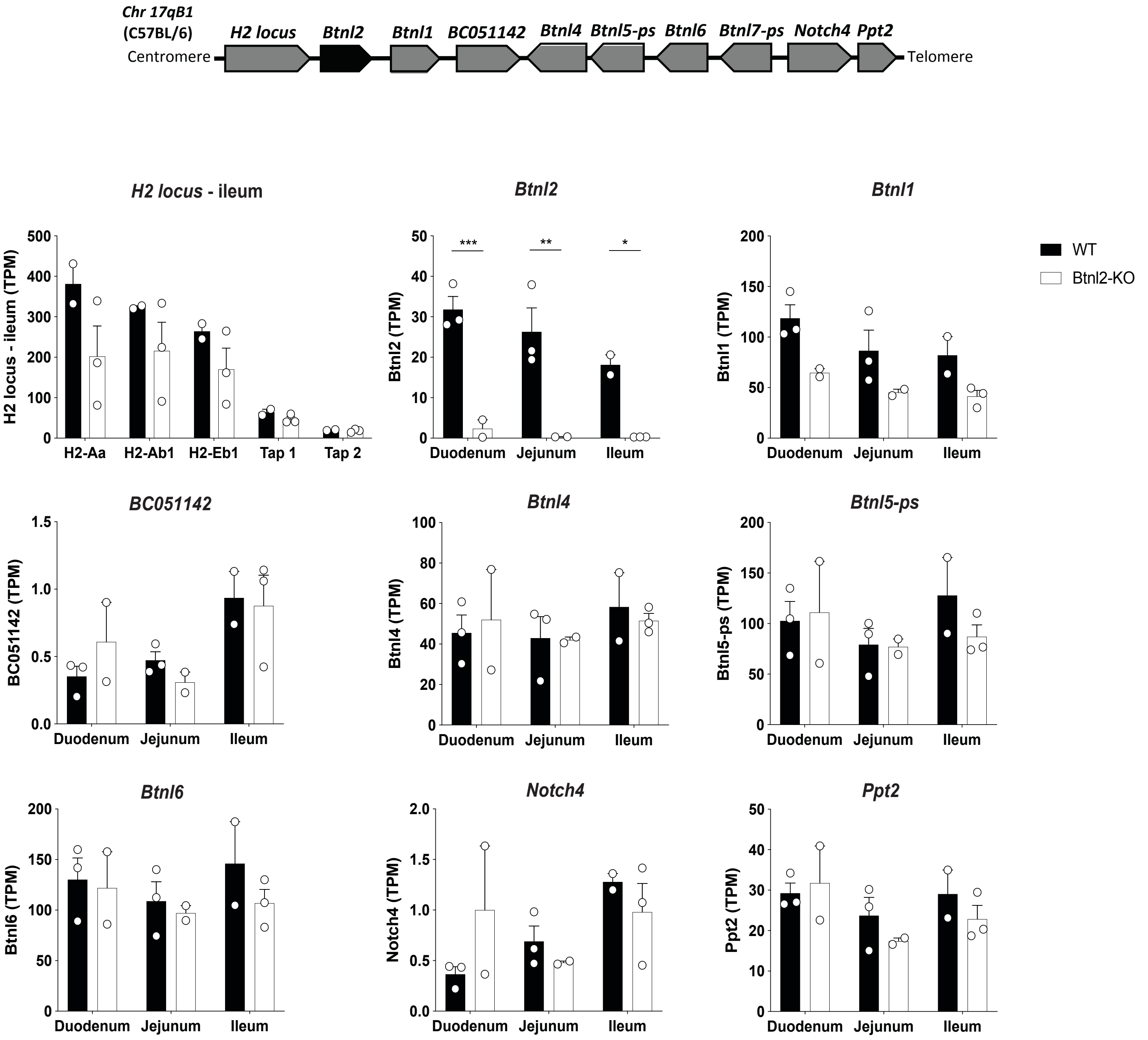

Supplementary Figure 1. Expression of Btnl2 neighboring genes is not significantly affected by Btnl2-LacZ deletion cassette.

Top panel - schematic representation of mouse Chr. 17qB1 locus based on USCS genome browser, showing the orientation and position of the genes near Btnl2 locus. Bottom panel - RNAseq reads (TPM) around the Btnl2 locus in intestinal epithelial cells isolated from different segments of the small intestine of cohoused 7-wk-old Btnl2-KO and WT mice (n=2-4, each). Error bars represent mean ± SEM.

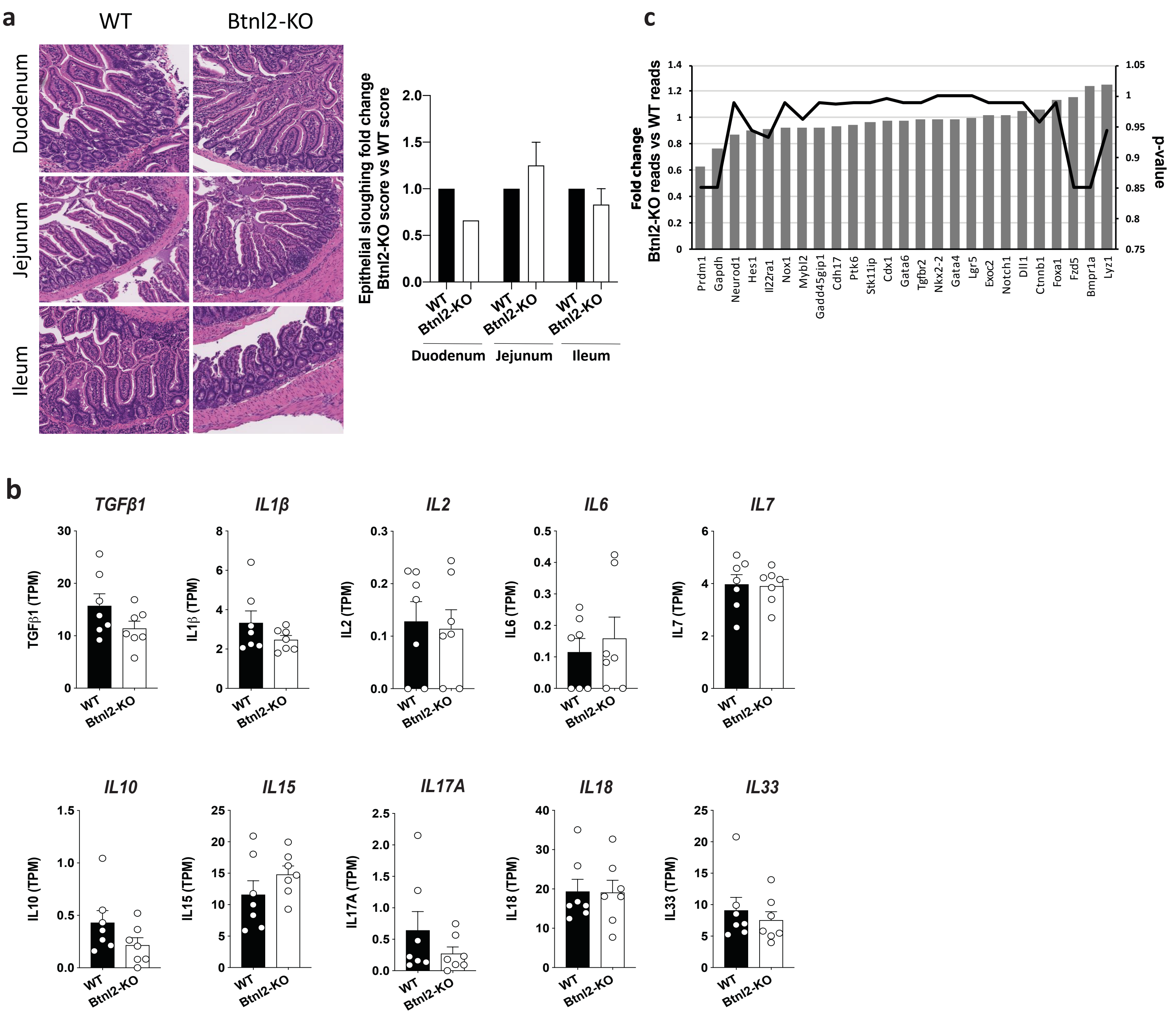

Supplementary Figure 2. Btnl2-KO mice do not develop spontaneous intestinal inflammation at steady-state. Duodenum, jejunum and ileum were collected from 7- and 17-wk-old cohoused Btnl2-KO and WT mice (n=4-7, each) and processed for RNAseq and histology. **(a)** Representative H&E staining and histological analysis of duodenum, jejunum and ileum of 17-wk-old Btnl2-KO and WT littermates. **(b)** Pro-inflammatory cytokines (TPM) in the ileum of 17-wk-old Btnl2-KO and WT littermates. Error bars represent mean ± SEM. **(c)** Intestinal epithelial cells (IECs) from jejunum/ileum of cohoused 7-wk-old Btnl2-KO and WT mice were processed for RNAseq. Plot represents fold change of Btnl2-KO reads relative to WT reads (grey bars) and their matching p-value (black line) in genes associated with differentiation and maturation of intestinal epithelial cells.

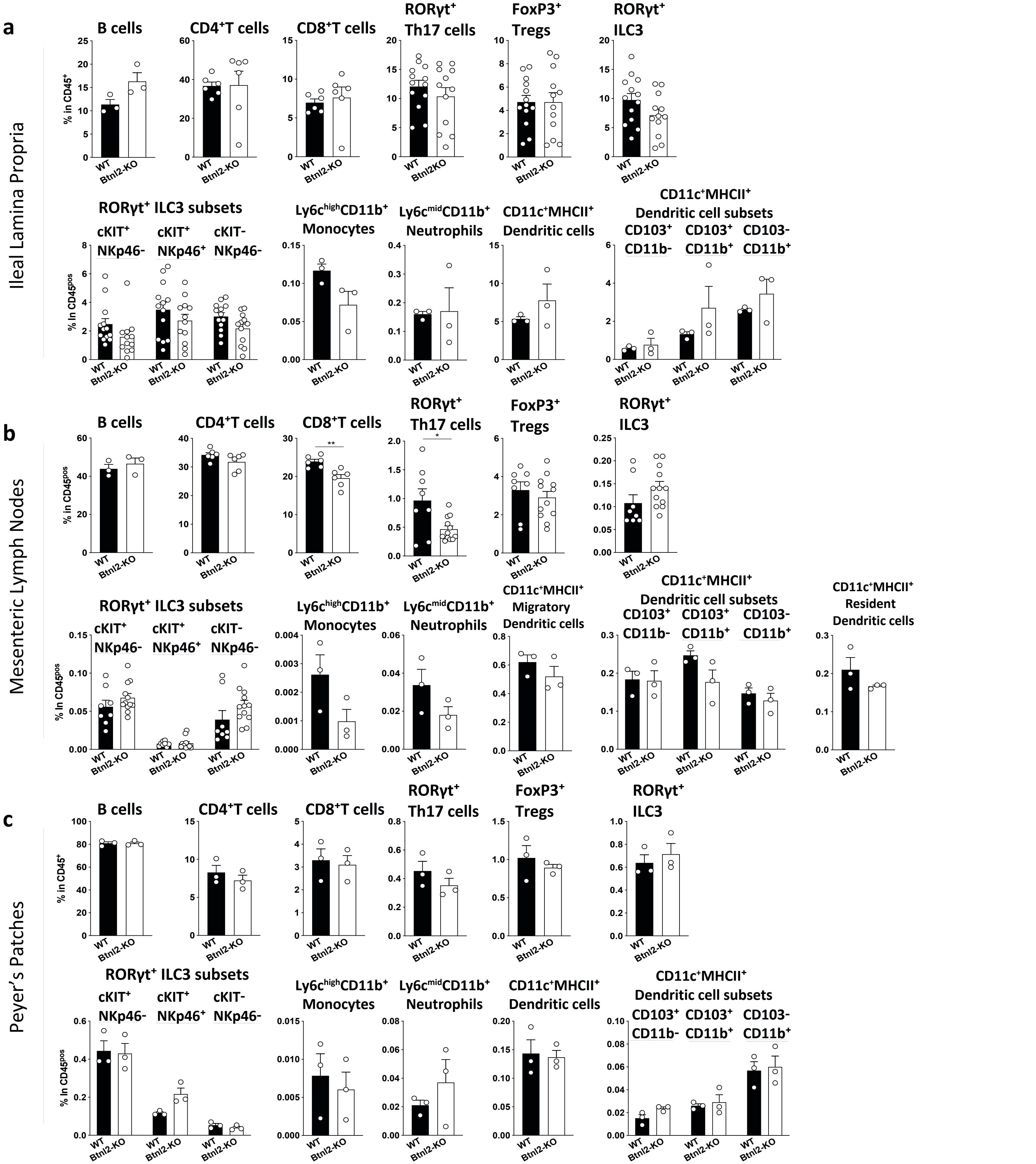

Supplementary Figure 3. Btnl2-KO mice exhibit comparable immune phenotypes in small intestine at steady state. Ileum, Peyer's Patches and mesenteric lymph nodes were collected from cohoused 7-17-wk-old Btnl2-KO and WT mice (n=3-13, each, pool of 1-3 separate experiments) and processed for flow cytometry. **(a-c)** Frequencies of major lymphocyte subsets within the ileal lamina propria **(a)**, mesenteric lymph nodes **(b)**, and Peyer's Patches **(c).** Error bars represent mean +/- SEM. Significance is measured using unpaired t-tests assuming similar SD, *p<0.05, **p<0.005, ***p<0.0005, significantly different from WT. Tregs, regulatory T cells. DCs, dendritic cells. ILC3, innate lymphoid cells type 3. Dendritic cells are gated on lineage (TCRβ, B220 and Ly6c) negative cells. ILC3 cells are also gated on lineage (CD11b, CD11c, Gr1, B220, TCRβ, and NK1.1) negative cells.

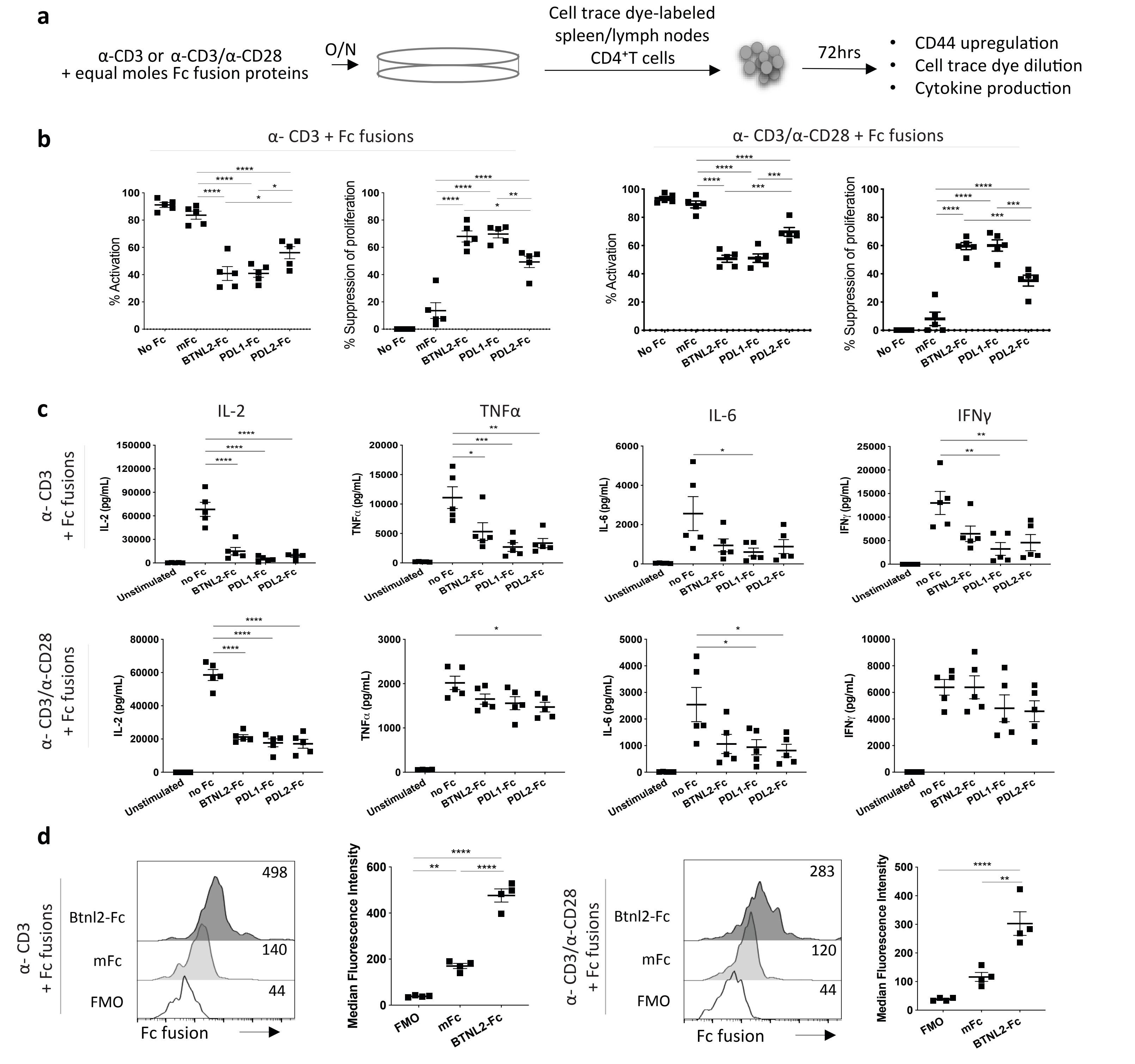

Supplementary Figure 4. Btnl2 suppresses activation and proliferation of CD4⁺T cells comparably to PDL1 and PDL2 *in vitro*.

Total CD4+T cells were enriched from the spleens and lymph nodes of 10-12-wk-old WT mice (n=5, each), labeled with CFSE and stimulated with α-CD3 or α-CD3/α-CD28 in the presence of different Fc fusions for 72hrs. At 72hrs, culture supernatants were collected, and cells were processed for flow cytometry. **(a)** Schematic design of in vitro CD4+T cell proliferation assay. **(b)** Graphs display activation and suppression of proliferation of CD4+T cells following 72hrs of culture. Activation is represented as the frequency of CD44+CD4+T cells. Suppression of proliferation calculated as the percent difference between the proliferation in the presence of a specific Fc fusion and no Fc fusion, relative to the proliferation in the absence of Fc fusion. **(c)** Levels of pro-inflammatory cytokines in supernatants derived from CD4+T cells cultures with α-CD3 or α-CD3/α-CD28 and equimolar concentrations of Fc fusion proteins for 72 hrs. **(d)** Histograms displaying putative Btnl2 receptor staining and median fluorescence intensity on activated CD4+T cells at 72hrs following α-CD3 or α-CD3/α-CD28 stimulation. Right panel - Median fluorescence intensity of Fc-fusion-stained CD4+T cells. Error bars represent mean +/- SEM. One-way ANOVA with Tukey's test for multiple comparisons, *p<0.05, **p<0.005, ***p<0.001, ****p<0.0001.

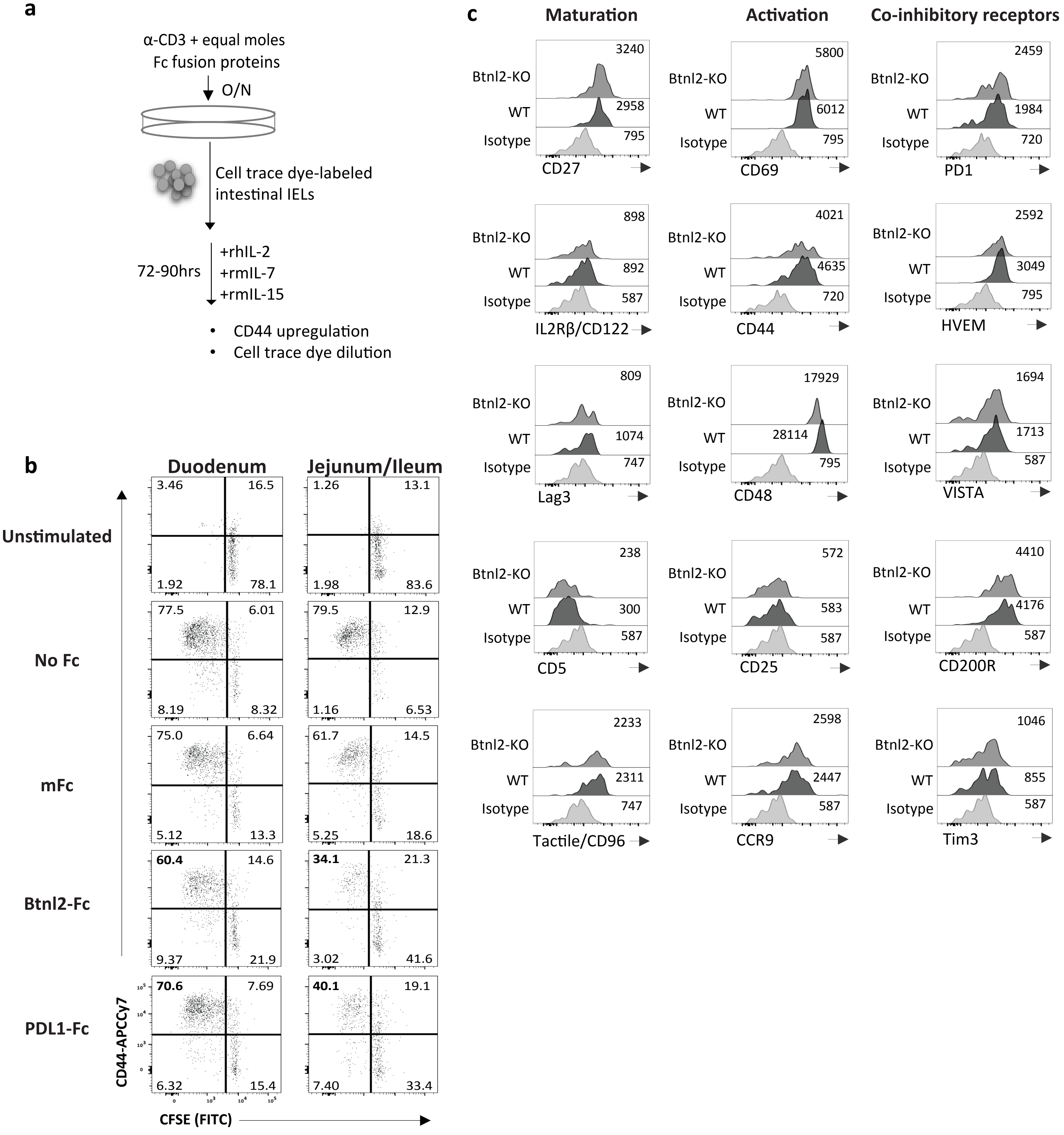

Supplementary Figure 5. Jejunal/Ileal Btnl2-KO and WT γδ IELs show comparable expression levels of coinhibitory receptors and markers of maturation and activation.

IELs were isolated from jejunum/ileum of cohoused 12-13-wk-old Btnl2-KO and WT (pool of 6 mice, each) and processed for flow cytometry by staining with LEGENDScreen mouse cell antibody panel (Biolegend, CA). Cells are gated on CD45+TCRβ-TCRγδ+CD8αα+. **(a)** Schematic design of in vitro IEL proliferation assay. **(b)** Representative flow cytometry plots of duodenal and jejunal/ileal Btnl2-KO CD8αα+ γδ IELs following 84hrs of culture in the presence of equimolar concentrations of Btnl2-Fc and control mFc fusion proteins. **(c)** Histograms of maturation, activation and co-inhibitory receptors for Btnl2-KO and WT CD8αα+ γδ IELs. MFI, mean fluorescence intensity.

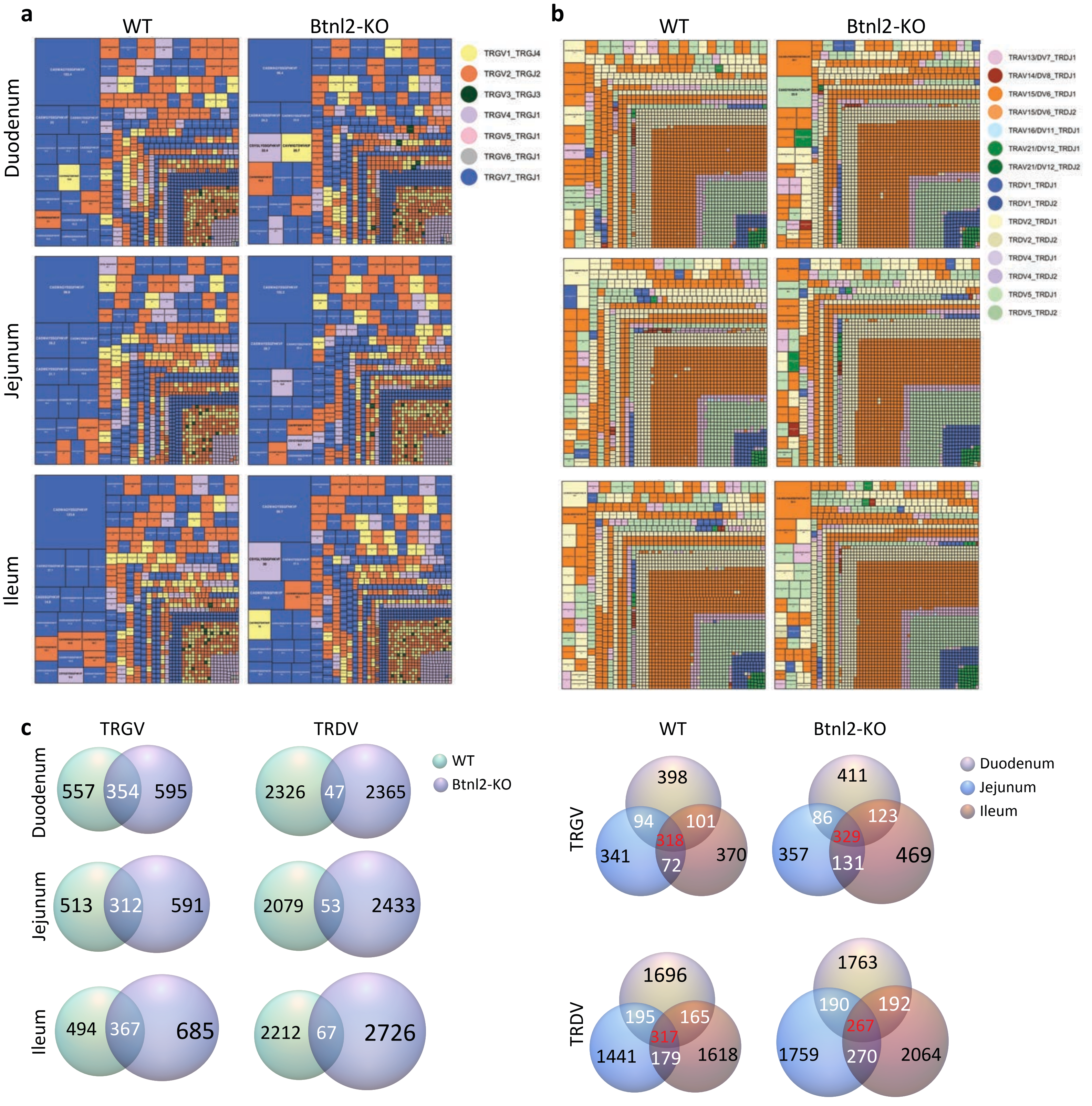

Supplementary Figure 6. Btnl2-KO γδ IELs have diverse TRGV and TRDV repertoire across small intestinal segments. γδ IELs from duodenum, jejunum and ileum of cohoused 11-wk-old Btnl2-KO and WT littermates (n=3-4/genotype, pool of 2 mice, each) were sort-purified as CD45+TCRβ-TCRγδ+ cells. Two thirds of each sample were processed for deep bulk RNA sequencing and one third of each sample was pooled per genotype per segment and used for single-cell sorting and single-cell RNA sequencing. **(a,b)** Treemap rendering of the all unique CDR3γ **(a)** or CDR3δ **(b)** aminoacid sequences/combined sample, in which the size of the box corresponds to the size of the CDR3γ or CDR3δ clone and the color of the box represents the TRGV/TRGJ or TRDV/TRDJ genes used. **(c)** Venn diagrams display the number of overlapping CDR3γ and CDR3δ clones per segment. **(d)** Venn diagrams display the number of overlapping CDR3γ and CDR3δ clones per genotype.

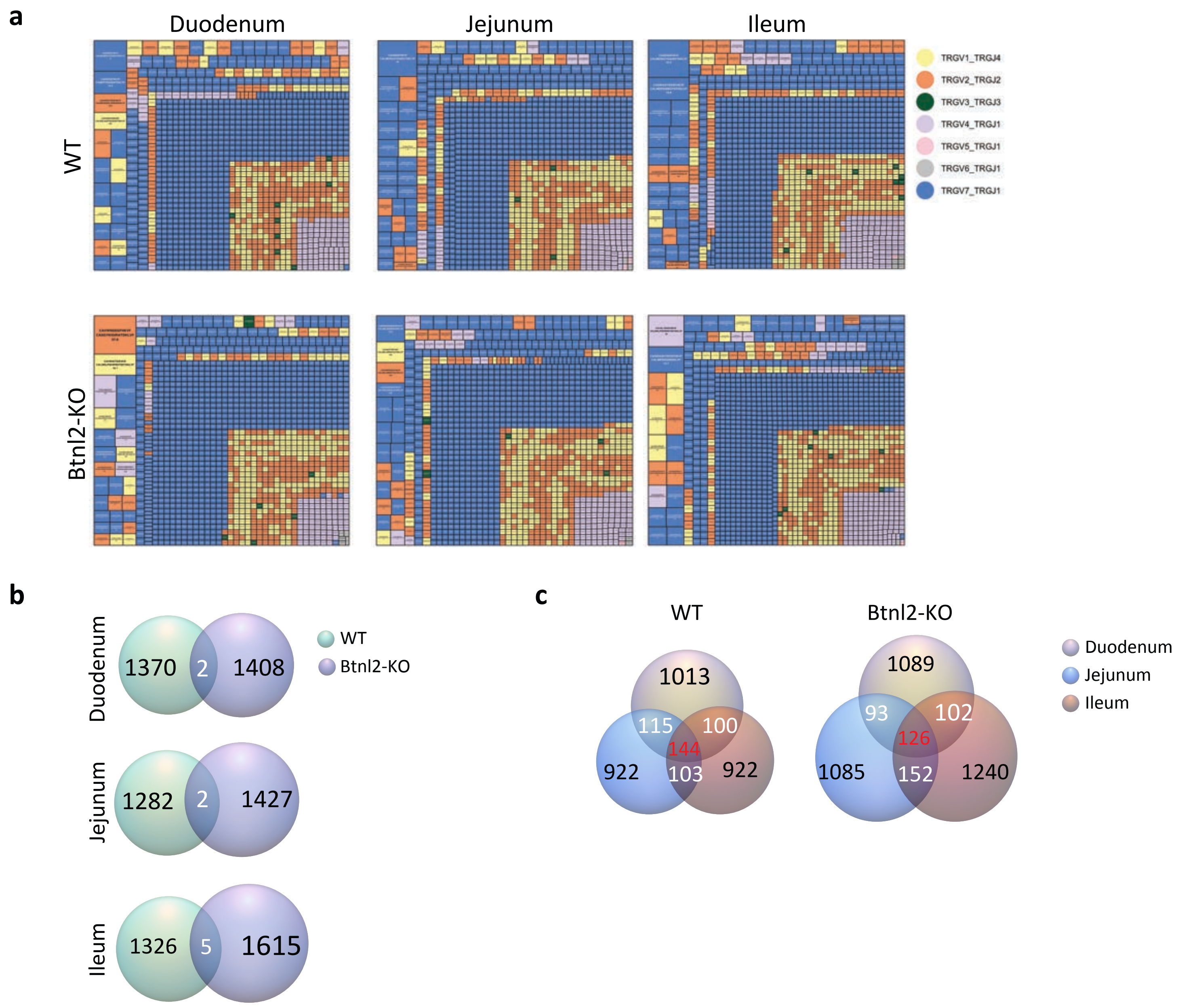

Supplementary Figure 7. CDR3γ/CDR3δ clone repertoire is unique to each small intestine segment and genotype. γδ IELs from duodenum, jejunum and ileum of cohoused 11-wk-old Btnl2-KO and WT littermates (n=3-4/genotype, pool of 2 mice, each) were sort-purified as CD45+TCRβ-TCRγδ+ cells. Two thirds of each sample were processed for deep bulk RNA sequencing and one third of each sample was pooled per genotype per segment and used for single-cell sorting and single-cell RNA sequencing. **(a)** Treemap rendering of all unique pairs of CDR3γ and CDR3δ aminoacid sequences/combined sample, in which the size of the box corresponds to the size of the paired CDR3γ/CDR3δ clone and the color of the box represents the TRGV/TRGJ genes used. **(b)** Venn diagrams display the number of overlapping paired CDR3γ/CDR3δ clones per segment. **(c)** Venn diagrams display the number of overlapping paired CDR3γ/CDR3δ clones per genotype.

a **b**

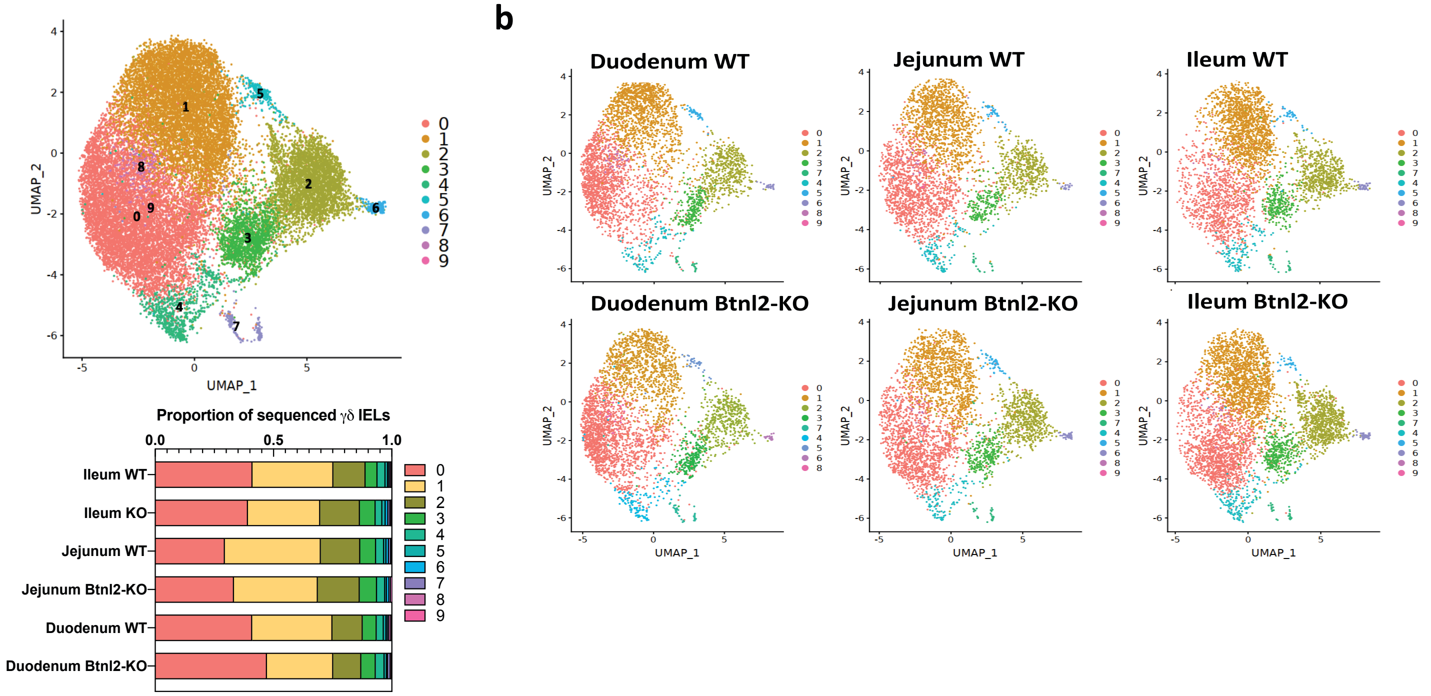

c

Cluster 0		Cluster 1		Cluster 2		Cluster 3		Cluster 4		Cluster 5		Cluster 6	
**Gene**	**Mean FC**	**Gene**	**Mean FC**	**Gene**	**Mean FC**	**Gene**	**Mean FC**	**Gene**	**Mean FC**	**Gene**	**Mean FC**	**Gene**	**Mean FC**
Tyrobp	5.63	Gzmk	2.86	Thy1	2.98	Xcl1	4.92	Ccl4	14.20	Isg15	7.87	Klf2	11.05
Il2rb	2.89	Scin	2.09	Tgm2	2.83	Capn3	2.81	Xcl1	7.42	Ifit1	4.61	Igfbp4	6.13
Cd200r2	2.48	Lat	1.78	Tcf7	2.53	Tcf7	2.46	Ccl3	5.33	Rtp4	4.09	Lef1	5.59
Cela1	2.35	Emp3	1.68	Ltb	2.45	Id3	2.44	Egr1	4.96	Irf7	3.99	Thy1	4.99
Fcer1g	2.23	Plac8	1.60	Rgs10	2.43	Kit	2.39	Nr4a1	4.83	Bst2	3.98	S1pr1	4.55
Lgals3	2.09	Epsti1	1.56	Id3	2.33	Cd160	2.37	Nr4a2	4.54	Stat1	3.48	CD8b1	4.38
Lag3	2.00	Hcst	1.45	Cd160	2.24	Ltb	2.16	Bcl2a1b	3.34	Zbp1	3.32	Rasgrp2	4.34
Gzma	1.92	Gzmb	1.45	Gm19705	2.19	Batf3	2.03	Tnfrsf9	3.26	Ifi27l2a	3.08	Ramp1	4.28
Itgad	1.78	Ccl5	1.45	Plac8	2.13	Mel1	2.02	Nfkbid	3.25	Ifit3	2.90	Sell	4.23
Cd7	1.75	Gadd45b	1.44	Ighm	2.09	Klrb1a	1.93	Egr2	3.05	Pydc3	2.79	Ms4a4b	4.08
Itih5	1.72	Itgb7	1.39	Ms4a4b	2.06	Gm156	1.77	Bcl2a1d	2.58	Ms4a4b	2.72	Socs3	4.00
Cd200r4	1.71	Cish	1.39	Tmsb10	2.04	1810041H14Rik	1.69	Tagap	2.57	Gbp6	2.61	Tagln2	3.52
Cd8a	1.70	Txnip	1.39	Ccr9	2.02	Clec2i	1.68	Nfkbia	2.55	Trim30a	2.53	Arl4c	3.33
Cd200r1	1.68	Dnajc15	1.38	Ly6e	1.98	Cox7a2	1.68	Mmd	2.25	Isg20	2.48	Ifi27l2a	3.30
Klrb1a	1.57	Mospd1	1.36	Jun	1.90	Nfkb1	1.67	Nr4a3	2.20	Xaf1	2.43	Rasa3	3.22
Tnfrsf9	1.56	Actn2	1.36	Tagln2	1.89	Ddit4	1.67	Irf8	2.16	Cmpk2	2.42	Dgka	3.11
Ptpn5	1.54	CD247	1.35	Rps19	1.87	Tmsb10	1.55	Zfp36l1	2.15	Lgals3bp	2.41	Tmsb10	3.07
Plek	1.54	Selm	1.35	Rps20	1.87	Evl	1.55	Egr3	2.14	Ms4a6b	2.36	Emp3	3.01
Epcam	1.52	Gmfg	1.35	Tmem108	1.84	Limd2	1.55	Rilpl2	2.13	Pydc4	2.32	Rgs10	2.99
Acot7	1.51	Anxa2	1.35	Evl	1.79	Zfp36l1	1.53	Ehd1	2.00	Rsad2	2.29	Dusp2	2.91

d

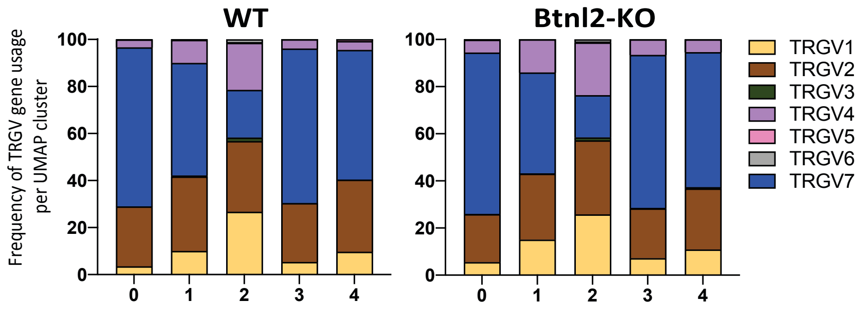

Supplementary Figure 8. γδ IELs can be subdivided into different clusters based on developmental origin, differentiation stage, and effector profile.

γδ IELs (CD45+TCRβ-TCRγδ+) from duodenum, jejunum and ileum of cohoused 11-wk-old Btnl2-KO and WT littermates (pool of 8 mice, each) were single-cell sorted and single cell RNA sequencing and single cell TCR sequencing were performed. (**a**) Combined UMAP plot with clusters defined by colors marking nine distinct clusters based on gene expression differences for >4400 cells/segment/genotype. Right panel - Proportion of sorted Btnl2-KO and WT γδ IEL single cells that segregate with each cluster. (**b**) Individual UMAP clustering of single cell RNA sequenced duodenal, jejunal and ileal Btnl2-KO and WT γδ IELs. (**c**) Top 20 genes and their mean Fold Change (FC) enrichment in clusters 0-7 relative to their expression in the whole single cell population. (**d**) Frequency of TRGV gene usage in different clusters in ileal Btnl2-KO and WT γδ IELs.

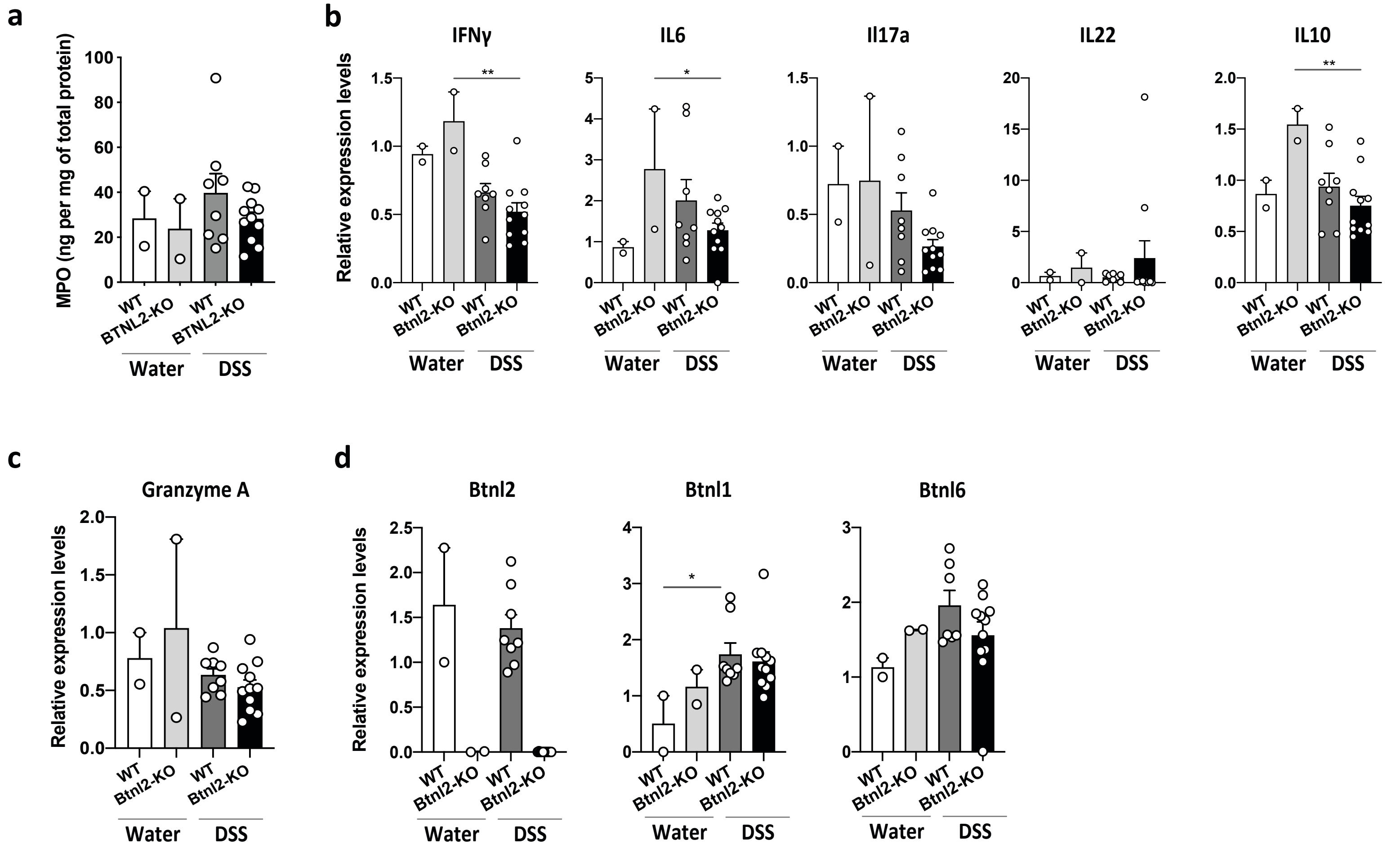

Supplementary Figure 9. Btnl2-KO mice exhibit comparable ileal immune responses to WT mice in chronic DSS-induced colitis.

Cohoused 15-wk-old Btnl2-KO (n=11) and WT (n=8) littermates were subjected to 3% DSS-induced colitis for 7 days followed by water for 8 days. Control mice (n=2-4) received water. **(a)** Myeloperoxidase (MPO) activity in ileal homogenates of water- and DSS-treated Btnl2-KO and WT mice. **(b)** Transcript levels of pro-inflammatory cytokines in ileal homogenates of water and DSS-treated Btnl2-KO and WT mice. Error bars represent mean +/- SEM. Significance is measured using one-way ANOVA, *p<0.05, **p<0.005. **(c)** Granzyme A mRNA levels in ileal homogenates of water- and DSS-treated Btnl2-KO and WT mice, normalized to β2m. **(d)** Btnl1/2/6 mRNA levels in ileal homogenates of water- and DSS-treated Btnl2-KO and WT mice, normalized to β2m.

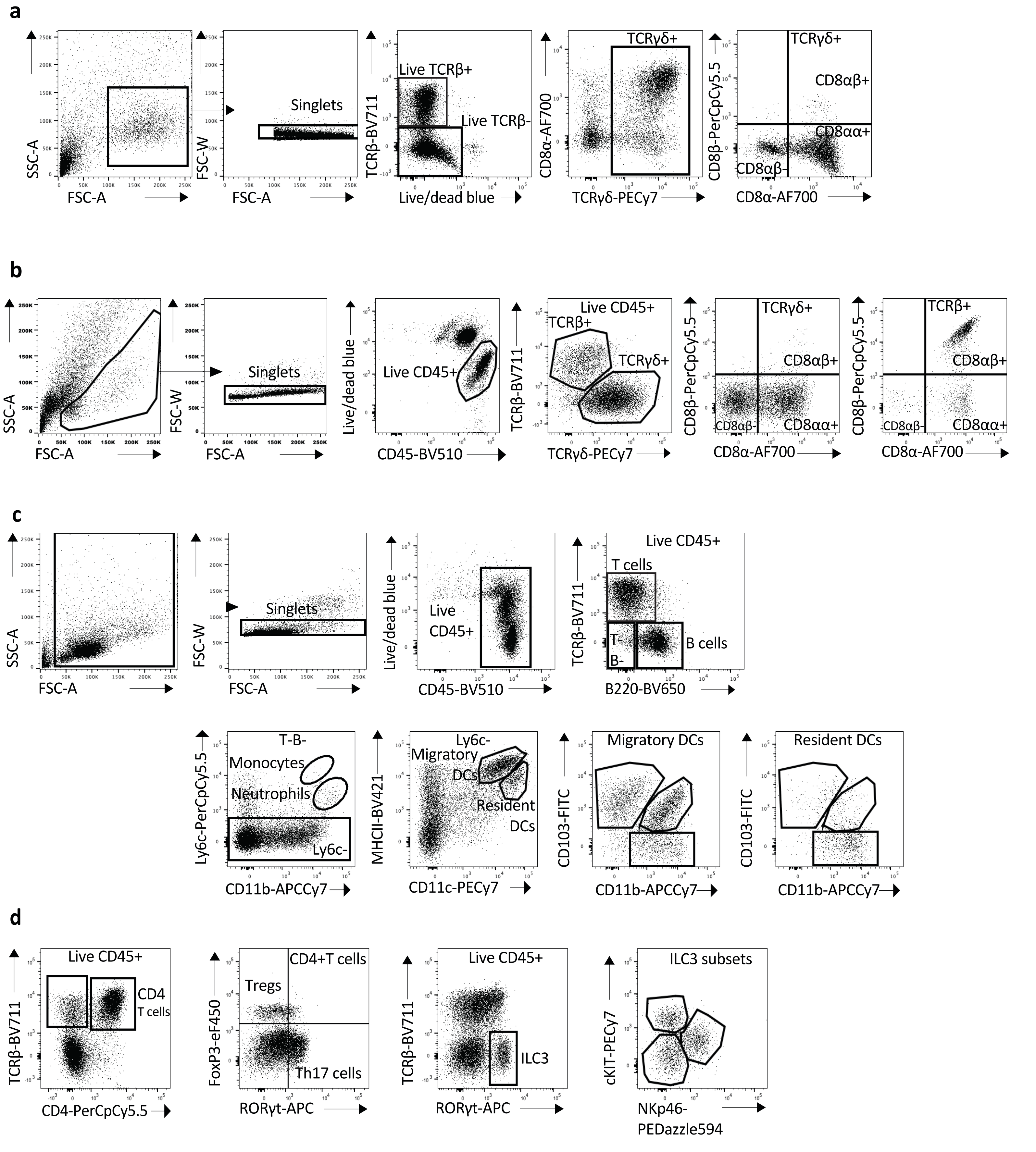

Supplementary Figure 10. Gating strategies for flow cytometry plots.

(**a**) Representative gating strategy for in vivo γδ IEL experiments, related to Figure 2a, d, Figure 3d. Example plots use an ileal IEL sample from a 12-wk-old Btnl2-KO mouse. (**b**) Representative gating strategy for in vitro IEL experiments, related to Figure 3a and Supplementary Figure 5b. Example plots use a duodenal IEL sample from a 12-wk-old WT mouse, cultured in the absence of Fc fusion proteins (no Fc). (**c-d**) Representative gating strategy for immune cell profiling of LPL, PP, and MLNs, related to Supplementary Figure 3. (**c**) Example plots use an MLN sample from a 12-wk-old WT mouse. (**d**) Example plots use an ileal LPL sample from a 12-wk-old WT mouse.
