# Supplementary material for: Butyrophilin-like 2 regulates site-specific adaptations of intestinal γδ intraepithelial lymphocytes
Source: Commun Biol. 2021 Jul 26;4:913. doi: 10.1038/s42003-021-02438-x (PMC8313535; doi:10.1038/s42003-021-02438-x)
Supplement: Supplementary file 2 — Description of Additional Supplementary Files [file 42003_2021_2438_MOESM2_ESM.pdf]

Description of Additional Supplementary Files

File name: Supplementary Data 1

Description: This document contains the source data for the graphs and charts from both main figures and supplementary figures. All information is contained within one excel file, with each tab representing a different figure.
