# Supplementary material for: Butyrophilin-like 2 regulates site-specific adaptations of intestinal γδ intraepithelial lymphocytes
Source: Commun Biol. 2021 Jul 26;4:913. doi: 10.1038/s42003-021-02438-x (PMC8313535; doi:10.1038/s42003-021-02438-x)
Supplement: Supplementary file 4 — Reporting Summary [file 42003_2021_2438_MOESM4_ESM.pdf]

nature research

Corresponding author(s): Sokol Haxhinasto

Last updated by author(s): Jun 14, 2021

Reporting Summary

Nature Research wishes to improve the reproducibility of the work that we publish. This form provides structure for consistency and transparency in reporting. For further information on Nature Research policies, see our Editorial Policies and the Editorial Policy Checklist.

Statistics

For all statistical analyses, confirm that the following items are present in the figure legend, table legend, main text, or Methods section.

n/a	Confirmed	
☐	☒	The exact sample size (*n*) for each experimental group/condition, given as a discrete number and unit of measurement
☐	☒	A statement on whether measurements were taken from distinct samples or whether the same sample was measured repeatedly
☐	☒	The statistical test(s) used AND whether they are one- or two-sided<br>*Only common tests should be described solely by name; describe more complex techniques in the Methods section.*
☐	☒	A description of all covariates tested
☐	☒	A description of any assumptions or corrections, such as tests of normality and adjustment for multiple comparisons
☐	☒	A full description of the statistical parameters including central tendency (e.g. means) or other basic estimates (e.g. regression coefficient) AND variation (e.g. standard deviation) or associated estimates of uncertainty (e.g. confidence intervals)
☐	☒	For null hypothesis testing, the test statistic (e.g. *F*, *t*, *r*) with confidence intervals, effect sizes, degrees of freedom and *P* value noted<br>*Give P values as exact values whenever suitable.*
☒	☐	For Bayesian analysis, information on the choice of priors and Markov chain Monte Carlo settings
☒	☐	For hierarchical and complex designs, identification of the appropriate level for tests and full reporting of outcomes
☒	☐	Estimates of effect sizes (e.g. Cohen's *d*, Pearson's *r*), indicating how they were calculated

Our web collection on statistics for biologists contains articles on many of the points above.

Software and code

Policy information about availability of computer code

Data collection

For both scRNAseq and bulk RNAseq: Following IEL isolation, cells were stained with LIVE/DEAD fixable blue dead cell stain as per manufacturer's instructions (Thermo Fisher Scientific). Fc receptors were blocked using purified anti-mouse CD16/32 (93) and 2% each of normal mouse serum, rat serum and hamster serum (Jackson Immuno Research) and cells were stained using the following antibodies: CD45-BV510 (30-F11), CD8α-AF700 (53-6.7), CD8β-PerCP/Cy5.5 (YTS156.7.7), TCRβ-BV711 (H57-597), TCRγδ-PE/Cy7 (GL3). Two mice were pooled per each sample. γδ IELs were sorted from each sample using MoFlo Astrios EQ (Beckman Coulter). Two thirds of each sample were resuspended in RNA Lysis Buffer (Zymo Research) and processed for bulk RNA sequencing. One third of each sample was pooled per segment of the small intestine per genotype, resuspended in PBS with 0.04% BSA and loaded on a Chromium Single Cell Instrument (10X Genomics). RNAseq and V(D)J libraries were prepared using Chromium Single Cell 5' Library, Gel Beads & Multiplex Kit (10X Genomics). After amplification, cDNA was divided into RNAseq and V(D)J library aliquots. To enrich the V(D)J library aliquot for γδ TCRs, cDNA was divided into two 10 ng aliquots and amplified in two rounds using internally designed primers. In particular, the following primers were used for the first round of amplification: MP147 for short R1 (ACACTCTTTCCCTACACGACGC), MP371 for mouse TRGC1-3 (/5Biosg/TTCCTGGGAGTCCAGGATRGTATTG), MP 372 for mouse TRGC4 (/5Biosg/CACCCTTATGACTTCAGGAAAGAACTTT) and MP369 for mouse TRDC (/5Biosg/TTCCACAATCTTCTTGGATGATCTGAG). For the second round of amplification, 20 ng aliquots from the first round were further amplified using MP147 for short R1 (ACACTCTTTCCCTACACGACGC), MP373 a nested R2 plus mouse TRGC(GTGACTGGAGTTCAGACGTGTGCTCTTCCGATCTGTCCCAGYCTTATGGAGATTTGT), and MP370 a nested R2 plus mouse TRDC (GTGACTGGAGTTCAGACGTGTGCTCTTCCGATCTTAGTCACCTCTTTAGGGTAGAAATCTT). V(D)J libraries were prepared from 25 ng of each mTRGC and mTRDC amplified cDNA. Paired-end sequencing was performed on Illumina NextSeq500 for RNAseq libraries (Read 1 26-bp for unique molecular identifier (UMI) and cell barcode, 8-bp i7 sample index, 0-bp i5, and Read 2 55-bp transcript read) and V(D)J libraries (Read 1 150-bp, 8-bp i7 sample index, 0-bp i5, and Read 2 150-bp read). For RNAseq libraries, Cell Ranger Single-Cell Software Suite (10X Genomics, v2.2.0) was used to perform sample de-multiplexing, alignment, filtering, and UMI counting. The mouse mm 10 genome assembly and RefSeq gene model for mouse were used for the alignment. For V(D)J libraries, Cell Ranger Single-Cell Software Suite (10X Genomics, v2.2.0) was used to perform sample de-multiplexing, de novo assembly of read pairs into contigs, align and annotate contigs against all the germline segment V(D)J reference sequences from IMGT, label and locate CDR3 regions, group clonotypes.

Data analysis

For scRNAseq: scRNAseq data were analyzed using Seurat R package. Cells with fewer than 500 genes or more than 10% of mitochondrial RNA content were excluded during the quality control (QC) step. The remaining cells underwent dimension reduction by PCA on the highly variable genes. Data were further reduced to the 2D space on the first 20 PCs using uniform manifold approximation and projection (UMAP). Cell clusters were determined using a graph-based unbiased clustering approach implemented in Seurat. Positive markers defining each cluster were identified using Wilcoxon rank sum test. Six representative markers were selected for each cluster to visualize in heatmaps.
For scTCRseq: After V(D)J sequences were assembled and annotated, only productive γ and δ TCR sequences were kept. Two TCR diversity metrics (i.e. species richness and exponential of Shannon entropy) were estimated for each sample using iNEXT R package. Species richness measured total unique clone numbers, whereas the Shannon index computed the uncertainty in predicting the identity of a sequence taken at random from the dataset. Both interpolated and extrapolated diversities were estimated, and 95% confidence interval was based on 50 bootstraps. TCR repertoires were visualized using Treemap R package (https://CRAN.R-project.org/package=treemap). Downstream TCR analysis such as V(D)J usage, shared TCR and integration of TCR and RNA-seq was performed using customized R scripts.

For bulk RNAseq: cDNA was synthesized and amplified (16-cycle PCR) from 5 ng total RNA using SMARTer® Ultra® Low RNA Kit (Clontech). Nextera XT library prep kit (Illumina) was used to generate the final sequencing library (12 PCR cycles performed to amplify libraries) using 1 ng of cDNA as the input. The amplified libraries were size-selected at 400~600 bp. Sequencing was performed on Illumina HiSeq®2500 (Illumina) by multiplexed paired-read run with 2X100 cycles. The sequencing reads were mapped to the customized mouse genome using ArrayStudio (OmicSoft). Sense-strand exon reads were used to quantify the gene expression level by RSEM algorithm implemented in ArrayStudio. Genes were flagged as detectable with minimum of 10 reads. Differential expressed gene analysis was performed using Deseq2. Genes with fold change | > 1.5 | and FDR < 0.05 were considered significantly differentially expressed. The differentially expressed genes were subjected to pathway enrichment analysis using Running Fish exact test in NextBio (www.nextbio.com). TCR hypervariable-region sequences were reconstructed using TRUST.

Graphs and statistics were prepared using GraphPad Prism8 and Microsoft Excel.

For manuscripts utilizing custom algorithms or software that are central to the research but not yet described in published literature, software must be made available to editors and reviewers. We strongly encourage code deposition in a community repository (e.g. GitHub). See the Nature Research guidelines for submitting code & software for further information.

Data

Policy information about availability of data

All manuscripts must include a data availability statement. This statement should provide the following information, where applicable:

- Accession codes, unique identifiers, or web links for publicly available datasets
- A list of figures that have associated raw data
- A description of any restrictions on data availability

The raw sequencing data can be accessed from GEO, the accession code is GSE178273.

Field-specific reporting

Please select the one below that is the best fit for your research. If you are not sure, read the appropriate sections before making your selection.

☒ Life sciences ☐ Behavioural & social sciences ☐ Ecological, evolutionary & environmental sciences

For a reference copy of the document with all sections, see nature.com/documents/nr-reporting-summary-flat.pdf

Life sciences study design

All studies must disclose on these points even when the disclosure is negative.

Sample size

No statistical methods were performed to determine sample size.
Sample sizes for individual studies were dependent on the genotype and age of the animals and these were indicated in the appropriate figure legends. Data was plotted as mean +/- SEM.

Data exclusions

No data were excluded.

Replication

All experiments were reproduced a minimum of 3 times with similar results.

Randomization

For acute DSS studies, animals were randomly assigned to water and DSS treatment groups. Overall, for both in vitro and in vivo experiments, regardless of the age of the animals, only cohoused wildtype and knockout female mice were used.

Blinding

Blinding was specifically used for scoring histopathology in the case of DSS studies. Otherwise, investigators designed, performed and analyzed all in vitro and in vivo experiments and thus, were not blinded. Every sample was analyzed and processed following the same criteria.

Reporting for specific materials, systems and methods

We require information from authors about some types of materials, experimental systems and methods used in many studies. Here, indicate whether each material, system or method listed is relevant to your study. If you are not sure if a list item applies to your research, read the appropriate section before selecting a response.

Materials & experimental systems

n/a	Involved in the study
☐	☒ Antibodies
☒	☐ Eukaryotic cell lines
☒	☐ Palaeontology and archaeology
☐	☒ Animals and other organisms
☒	☐ Human research participants
☒	☐ Clinical data
☒	☐ Dual use research of concern

Methods

n/a	Involved in the study
☒	☐ ChIP-seq
☐	☒ Flow cytometry
☒	☐ MRI-based neuroimaging

Antibodies

Antibodies used

CD45-BV510 (Biolegend, Clone#30-F11, Cat#103138),
CD8α-AF700 (Biolegend, Clone#53-6.7, Cat#100730),
CD8β-PerCP/Cy5.5 (Biolegend, Clone#YTS156.7.7, Cat#126610),
TCRβ-BV711 (Biolegend, Clone#H57-597, Cat#109243)
TCRβ-APC/Cy7 (Biolegend, Clone#H57-597, Cat#109220),
TCRγ/δ-PE/Cy7 (Biolegend, Clone#GL3, Cat#118124),
CD11b-APC/Cy7 (Biolegend, Clone#M1/70, Cat#101226),
CD11c-APC/Cy7(Biolegend, Clone#N418, Cat#117324)
CD11c-AF700 (Biolegend, Clone#N418, Cat#117320),
CD11c-PE/Cy7 (Biolegend, Clone#N418, Cat#117318),
Gr1-APC/Cy7 (Biolegend, Clone#RB6-8C5, Cat#108424),
B220-APC/Cy7 (Biolegend, Clone#RA3-6B2, Cat#103224)
B220-AF700 (Biolegend, Clone#RA3-6B2, Cat#103232)
B220-BV650 (Biolegend, Clone#RA3-6B2, Cat#103241),
NK1.1-APC/Cy7 (Biolegend, Clone#PK136, Cat#108724),
MHCII-BV421 (Biolegend, Clone#M5/114.15.2, Cat#107632),
Ly-6C-PerCP/Cy5.5 (Biolegend, Clone#HK1.4, Cat#128012),
CD64-PE (Biolegend, Clone#X54-5/7.1, Cat#139304),
CD103-FITC (Biolegend, Clone#2E7, Cat#121420),
CX3CR1-Biotin (Biolegend, Clone#SA011F11, Cat#149018),
Streptavidin-PE/Dazzle 594 (Biolegend, Cat#405248),
NKp46-PE/Dazzle594 (Biolegend, Clone#29A1.4, Cat#137630),
CD4-PerCP/Cy5.5 (Biolegend, Clone#GK1.5, Cat#100434),
CD4-VF450 (Tonbo Biosciences, Clone#GK1.5, Cat#75-0041-U100),
c-KIT-PE-Cy7 (Biolegend, Clone#ACK2, Cat#135112),
RORγt-APC (eBiosciences, Clone#AFKJS-9, Cat#17-6988-82),
FoxP3-AF700 (eBioscience, Clone#FJK-16s, Cat#56-5773-82),
FoxP3-eF450 (eBioscience, Clone#FJK-16s, Cat#48-5773-82),
CD44-APC/Cy7 (Biolegend, Clone#IM7, Cat#103028),
CD44-BV650 (Biolegend, Clone#IM7, Cat#103049),
CD16/32 (Biolegend, Clone#93, Cat#101302),
CD16/32 (BD Pharmigen, Clone#2.4G2, Cat#553142),

BrdU-AF647 (Thermo Fisher Scientific, Clone# MoBU-1, Cat#B35133)
CellTrace CFSE Cell Proliferation dye (Thermo Fischer Scientific, Cat#C34554)
LIVE/DEAD fixable blue dead cell stain (Thermo Fischer Scientific, Cat#L23105)
LegendScreen Mouse PE kit (Biolegend, Cat#700005)

Validation

All antibodies were either previously published or validated by the commercial manufacturers (BD Biosciences, eBiosciences, Biolegend, ThermoFisher Scientific) and by Regeneron Pharmaceuticals Flow Cytometry Core.

Animals and other organisms

Policy information about studies involving animals; ARRIVE guidelines recommended for reporting animal research

Laboratory animals

Eight- to twelve-week-old female C57BL/6 mice were obtained from Jackson Laboratory. Btnl2-KO mice on a C57BL/6 background were generated and maintained at Regeneron Pharmaceuticals Inc. using the VelociGene technology. Btnl2-KO and WT female mice were used at 10-17 weeks of age for all the experiments except when otherwise indicated. Female littermates were cohoused after weaning for several weeks and assigned randomly to experimental groups in disease settings.

Wild animals

No wild animals were used in this study.

Field-collected samples

No field-collected samples were used.

Ethics oversight

All animals were maintained under pathogen-free conditions and experiments were performed according to protocols approved by

Ethics oversight | the Institutional Animal Care and Use Committee at Regeneron Pharmaceuticals Inc.

Note that full information on the approval of the study protocol must also be provided in the manuscript.

Flow Cytometry

Plots

Confirm that:

☒ The axis labels state the marker and fluorochrome used (e.g. CD4-FITC).

☒ The axis scales are clearly visible. Include numbers along axes only for bottom left plot of group (a 'group' is an analysis of identical markers).

☒ All plots are contour plots with outliers or pseudocolor plots.

☒ A numerical value for number of cells or percentage (with statistics) is provided.

Methodology

Sample preparation	To isolate IELs and LPLs: The small intestine was divided into three equal segments and lymphocyte isolation proceeded as described previously. Briefly, to isolate IEC and IEL fractions, small intestine was cut into 2 cm pieces and incubated in HBSS containing 5mM EDTA, 10mM HEPES and 2% fetal calf serum (FCS) twice for 15 min at 37?C with shaking at 150 rpm. After vigorous vortexing, the intestinal pieces were washed over 100 µm cell strainer and centrifuged on a 40%/80% Percoll gradient (GE Healthcare) at 2500 rpm for 20 min at 20oC. The top layer containing IECs was collected, washed and resuspended in Trizol for RNA extraction. IEL fraction was collected from the interface, washed and resuspended in Miltenyi MACS buffer. Following IEL isolation, LPLs were isolated from intestinal pieces by incubation in HBSS w/o Ca2+/Mg2+ supplemented with 50 U/mL Collagenase D (Roche), 0.25 mg/mL DNase I (Sigma-Aldrich), 50 U/mL Dispase (Corning) and 5% FCS for two rounds of 25 min at 37C with shaking at 150 rpm. Cells were centrifuged on a 40%/80% Percoll gradient (GE Healthcare) and LPLs were collected from the interface, washed and resuspended in MACS buffer for immediate surface cell staining.<br>To isolate immune cells from MLNs and PPs: Peyer's Patches were collected from the whole small intestine, washed with ice-cold DPBS and incubated with 50 U/mL Collagenase D (Roche), 0.25 mg/mL DNase I (Sigma-Aldrich), 50 U/mL Dispase (Corning) and 5% FCS for 25 min at 37?C with shaking at 150 rpm. Mesenteric lymph nodes were minced in HBSS with Ca2+/Mg2+containing 15 U/mL Collagenase D (Roche) and 50 µg/mL DNase I (Sigma-Aldrich), and incubated for 20 min at 37?C without shaking. Cells were resuspended in MACS buffer for immediate surface staining.
Instrument	LSR Fortessa X-20
Software	Samples were collected on BD FACSDiva, analyzed on FlowJo V10 and statistical tests were performed using GraphPad Prism8 and Microsoft Excel.
Cell population abundance	Gamma delta IELs were sort-purified for RNAseq and TCRseq experiments. Abundance was dependent on the small intestinal segment of origin. Specifically, gamma delta IELs represented cca 60% of live CD45 positive cells in the duodenum IEL compartment; cca 45% of live CD45 positive cells in the jejunum IEL compartment; and cca 30% of live CD45 positive cells in the ileal IEL compartment. Overall, live CD45 positive cells comprised up to 35% of the freshly isolated IEL fraction. The remaining, CD45 negative, live fraction consisted of intestinal epithelial cells.

Gating strategy

For in vitro IEL experiments: Cells were first gated to remove cell debris using SSC-A, FSC-A (lymphocytes); then singlets were gated using FSC-W, FSC-A; then live dead negative cells were gated (viable cells); then CD45 positive cells were gated (live CD45 positive cells); then gamma delta T cells were gated on from a TCRbeta vs TCRgd gate (TCRb+ or TCRgd+); and CD8alpha vs CD8beta populations were gated either within the TCRgd gate or the TCRb gate (CD8aa+, CD8ab+, CD8ab-).

For in vivo IEL experiments: Cells were first gated to remove cell debris using SSC-A, FSC-A (lymphocytes); then singlets were gated using FSC-W, FSC-A; then live dead negative cells were gated (viable cells); then gamma delta T cells were gated from a TCRbeta vs TCRgd gate via exclusion of TCRb positive cells (TCRb- gate); and CD8alpha vs CD8beta populations were gated either within the TCRgd gate or the TCRb gate (CD8aa+, CD8ab+, CD8ab-).

For all other populations isolated from LPL, MLN and PP: Cells were first gated to remove cell debris using SSC-A, FSC-A (lymphocytes); then singlets were gated using FSC-W, FSC-A; then live dead negative cells were gated (viable cells); then CD45 positive cells were gated (live CD45 positive cells); then gates specific for the populations were created as follows:
within TCRbeta vs B220 (3 gates - B220+ = B cells; TCRbeta+ = total T cells; TCRb-B220- = myeloid/granulocyte populations);
within TCRbeta+ gate, CD4 vs CD8a (2 gates - CD4+ = CD4+ T cells; CD8a+ = CD8+ T cells);
within CD4+ T cell gate, RORgammat vs Foxp3 (2 gates - RORgammat+FoxP3- = Th17 cells; RORgammat-FoxP3+ = FoxP3+ Tregs);
within TCRb-B220- gate, Ly6C vs CD11b (3 gates- Ly6Chi CD11b+ = monocytes; Ly6Cmid CD11b+ = neutrophils; Ly6C- = all other myeloid populations);
within Ly6C- gate, MHCII vs CD11c (1 gate for LPL, PP - MHCII+CD11c+ = dendritic cells; 2 gates for MLN - MHCIIhiCD11c+ = migratory dendritic cells; MHCIImidCD11c+ = resident dendritic cells);
within MHCII+CD11c+ gate, CD11b vs CD103 (3 gates - CD11b-CD103+, CD11b+CD103+, CD11b+CD103-).

For ILC3, following gating on live CD45 positive cells, lineage positive cells were removed with a dump channel (CD11b, CD11c, Gr1, B220, and NK1.1) followed by TCRbeta vs RORgammat. ILC3 cells were gated as RORgammat+TCRbeta-. Within ILC3, subsets were gated with cKIT vs NKp46 (3 gates - cKIT+NKp46-, cKIT+NKp46+, cKIT-NKp46-).

☒ Tick this box to confirm that a figure exemplifying the gating strategy is provided in the Supplementary Information.
